# Epidermal Growth Factor Signalling Controls Myosin II Planar Polarity to Orchestrate Convergent Extension Movements during *Drosophila* Tubulogenesis

**DOI:** 10.1371/journal.pbio.1002013

**Published:** 2014-12-02

**Authors:** Aditya Saxena, Barry Denholm, Stephanie Bunt, Marcus Bischoff, Krishnaswamy VijayRaghavan, Helen Skaer

**Affiliations:** 1 Department of Zoology, University of Cambridge, Cambridge, United Kingdom; 2 School of Biology, St Andrews, Scotland, United Kingdom; 3 National Centre for Biological Sciences GKVK Campus, Bangalore, Karnataka, India; Institut Curie, France

## Abstract

Most epithelial tubes arise as small buds and elongate by regulated morphogenetic processes including oriented cell division, cell rearrangements, and changes in cell shape. Through live analysis of *Drosophila* renal tubule morphogenesis we show that tissue elongation results from polarised cell intercalations around the tubule circumference, producing convergent-extension tissue movements. Using genetic techniques, we demonstrate that the vector of cell movement is regulated by localised epidermal growth factor (EGF) signalling from the distally placed tip cell lineage, which sets up a distal-to-proximal gradient of pathway activation to planar polarise cells, without the involvement for PCP gene activity. Time-lapse imaging at subcellular resolution shows that the acquisition of planar polarity leads to asymmetric pulsatile Myosin II accumulation in the basal, proximal cortex of tubule cells, resulting in repeated, transient shortening of their circumferential length. This repeated bias in the polarity of cell contraction allows cells to move relative to each other, leading to a reduction in cell number around the lumen and an increase in tubule length. Physiological analysis demonstrates that animals whose tubules fail to elongate exhibit abnormal excretory function, defective osmoregulation, and lethality.

## Abstract 2

A study in fruit flies shows that during the elongation of embryonic renal tubules, graded signalling provides axial information for polarized myosin pulses that shorten cells circumferentially, driving intercalation of the cells and elongation of the tubule.

## Author Summary

Many of the tissues in our bodies are built up around complex arrays of elongated cellular tubes, which permit the entry, exit, and transport of essential molecules such as oxygen, glucose, and water. These tubes often arise as short buds, which elongate dramatically as the organ grows. We sought to understand the mechanisms that govern such transformations of shape using the fly renal tubule as a model. We find that elongation of this tissue is predominantly driven by cell rearrangement. Cells move around the circumference of the tubule, intercalating with each other so that the cell number around the lumen reduces, while increasing along the length of the tube. Our next question was how cells sense the direction in which they should move. We show that cells orient their position in the tissue by reading a signal sent out by a specific pair of cells at the tip of each tube. Cells use this directional information to make polarised movements through the asymmetric activity of the cell's contractile machinery. We find that the activity of myosin—the motor protein that regulates contraction—is pulsatile and polarised within the cell. This activity shortens the cells' circumferential lengths, so that cells move past each other around the tube circumference, thereby intercalating and producing tube elongation. We go on to show that excretory physiology is severely impaired when elongation fails, underlining the importance of sculpting organs with appropriate dimensions.

## Introduction

Our tissues and organs are built up around arrays of tubes that allow the exchange of nutrients, ions, and gases vital for bodily function. These tubules have precise architectures tailored to their physiological activities. It is important that appropriate tubule dimensions are established during development and maintained throughout life and where this fails, as for example in human polycystic kidney diseases, in which nephron diameters are grossly enlarged [1], physiological function is severely compromised, often leading to organ failure.

**Figure 1:**
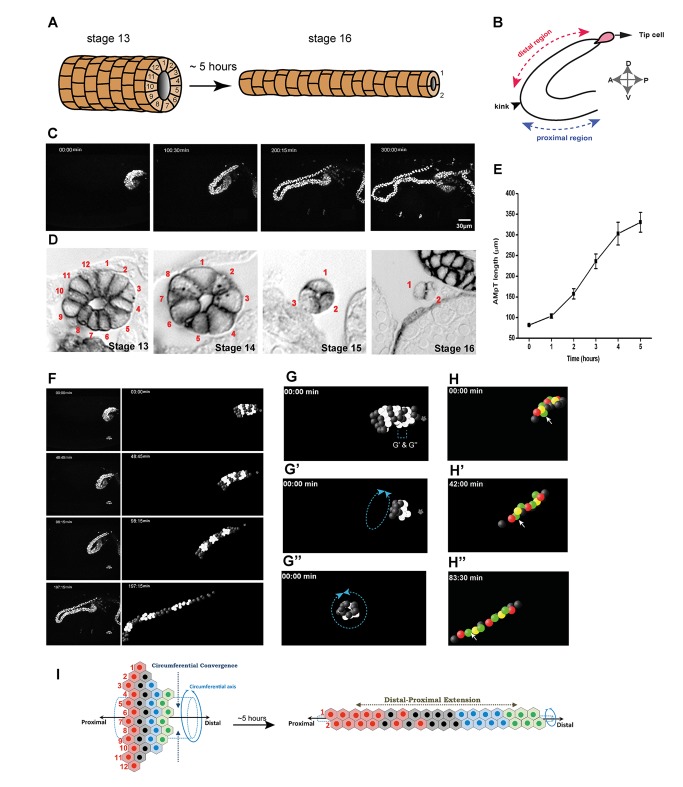
Convergent-extension movements drive MpT elongation. (A) Cartoon depicting embryonic MpT morphogenesis. At stage 13, MpTs are short, thick single-layered epithelial tubes with up to 12 cells surrounding the central lumen. Over 5–6 hours, the tubules elongate by cell rearrangement until just two cells surround the lumen. (B) Diagram showing the distal (red arrow) and proximal (blue arrow) aMpT regions, with the TC (pink) at the distal end. Arrowhead indicates the “kink” region. A-P, anterior-posterior; D-V, dorso-ventral embryonic axes. (C) Time-lapse sequence showing the morphogenesis of a *ctB>Stinger::dsRed* aMpT (Movie S1). (D) Transverse sections of embryonic aMpTs stained with anti-FasII (stages 13–16). Sections taken from the distal region show that the number of cells surrounding the lumen decreases from 12 to 2. (E) MpTs increase in length from 82.1±0.8 µm at stage 13 to 330.6±24.2 µm 5 hours later (stage 16) (*n* = 4 aMpTs from four different embryos; ± standard error of the mean [SEM]). (F) Left panels, stills from live imaged *ctB>Stinger::dsRed* aMpT (Movie S2) with 4-D reconstructions of distal region (right panels). Positions of tubule nuclei are represented as spheres. Circumferential rings of cells (black/white) rearrange into proximo-distally adjacent groups of cells by the end of tubule elongation. (G–G″) rotation of the tubule through 90° demonstrates the circumferential arrangement (two rings of cells, Movie S3). Star, TC. (H) 4-D reconstruction of a few distal cells in the top plane of an aMpT. Arrows indicate a cell (green) that intercalates between yellow and red neighbours. (I). Cartoon of MpT epithelium as a 2-D sheet. As the tubule elongates in the distal-proximal axis, circumferential neighbours intercalate.

Many tissues are sculpted during development by convergent extension (CE) movements. This process describes the concomitant narrowing of a tissue in one axis while it elongates along a perpendicular axis (Figure 1A) [2]–[4]. CE is brought about by changes in cell-neighbourhood relationships produced by cell intercalation. These changes can be driven by a variety of force-generating processes, such as lamellipodial protrusion, that allow cells to crawl over one another [5] or by cell-junction remodelling [5]–[7]. In both cases cell intercalation is highly organised and is polarised in the plane of the tissue [2],[8].

The insect renal or Malpighian tubules (MpTs) eliminate metabolic and foreign toxins and maintain the animal's ionic, acid-base, and water balance [9],[10]. They are long, narrow, single cell-layered epithelial tubes with a distinct distal-to-proximal (D-P) axis in which the distal regions are secretory in function and proximal regions have reabsorptive roles [11]. In *Drosophila* the tubules evert from the embryonic hindgut as short buds. During mid-embryogenesis they undergo a dramatic transformation in a period of just a few hours—increasing in length approximately 4-fold whilst narrowing substantially around their circumference. Tubule extension occurs in the absence of cell division and is accompanied by substantial rearrangement of cells within the plane of the epithelium [12]. This morphological transformation appears to be a dramatic example of CE and, because it occurs in the absence of cell proliferation that might complicate analysis, it is an attractive model to study the process of CE and its regulation.

How CE is controlled at the tissue level is still poorly understood in terms of the mechanisms and signals that orchestrate local cell behaviours to bring about orderly morphogenesis in the tissue as a whole. During *Drosophila* germband extension the segmentation genes that pattern the anterior-posterior axis are important in establishing planar polarity [13]. However, it is not known whether the influence of the segmentation genes is direct, nor have the mechanisms by which these genes control cell intercalation been established [4],[14],[15]. In other tissues the core planar cell polarity (PCP) genes regulate both oriented cell divisions [16]–[19] and polarised cell movements that underlie tissue extension [20]–[22], but details of the mechanisms involved remain elusive [23],[24].

Here we address the fundamental question of how cell intercalation is controlled at the tissue level, using the developing fly renal system as a model. We analyse cell movements during tubule extension and show that elongation results from circumferential cell intercalation associated with pulsatile and planar polarised accumulation of the motor protein, Myosin II in the basal cortex of cells. We consider the spatial cues that direct these oriented cell intercalation events as the tubule lengthens. We show that a polarised signal within the tubule organises cell rearrangement during CE. Using a combination of genetic manipulation, laser ablation, and live imaging we provide evidence that the epidermal growth factor (EGF) pathway ligand Spitz provides this cue. Spitz is expressed in the distal tubule tip, activating graded EGF signalling along the tubule, which is required for coordinated cell intercalation. EGF signalling acts to establish an axis of planar polarity in tubule cells at the onset of cell intercalation, which is independent of the activity of planar polarity genes. Perturbation of EGF signalling results in disorganised Myosin II dynamics, failure of cell intercalation, and defective elongation, leading to impaired tubule function and the failure of fluid homeostasis.

## Results

### Convergent Extension Movements Shape MpTs during Embryonic Development

By mid embryogenesis (stage 13) the tubules are short and stubby in shape, measuring approximately 80 µm in length (Figure 1A, 1C, and 1E) with between 10 and 12 cells surrounding the lumen (Figure 1A and 1D). Over approximately 5 hours, they undergo a 4-fold elongation to approximately 320 µm in length (Figure 1A, 1C, and 1E; Movie S1) whilst the number of cells surrounding the lumen reduces progressively to just 2 cells (Figure 1D). Imaging this morphogenetic transformation in real time and tracking individual cells reveals that rings of cells around the tubule lumen (as shown in Figure 1G′ and 1G″) intercalate circumferentially, becoming more spread out in the orthogonal, distal-to-proximal axis (Figures 1F and 1G; Movies S2 and S3). Cell intercalations can be followed (Figure 1H and 1H″; Movies S2 and S4), and we were able to confirm previous observations that tubule extension occurs sequentially with CE occurring in the distal half of the tubule earlier than in the proximal half [25]. Focussing on the distal region of the anterior tubules shown in Figure 1B and 1F we found that individual intercalation events took between 24 and 49 minutes, with an average of 42.2 min (±6.1 min, *n* = 4 intercalations) with cells moving at an average of 1.14 µm min^−1^ (±0.02 µm min^−1^, *n* = 9 cells). Our observations confirm the hypothesis that tubule elongation results primarily from oriented CE movements, in which cell intercalation around the circumferential axis produces orthogonal extension in the D-P axis of the tubule (Figure 1I).

### Control of CE Movements Is Intrinsic to the Tubule and Requires the Tip Cell Lineage

**Figure 2:**
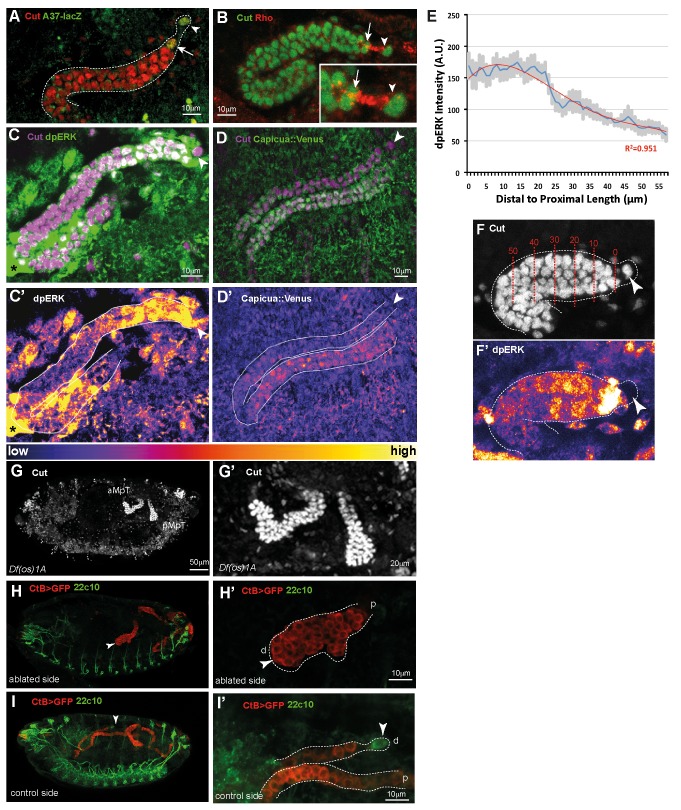
The tip cell lineage is required for tubule elongation. (A) Stage 14 aMpT stained with anti-Cut (red) with TC (arrowhead) and SC (arrow) highlighted with Neuromusculin-LacZ (A37, green). (B) Stage 14 aMpT stained with anti-Cut (green) and anti-Rhomboid (red) showing Rhomboid in tip (arrowhead) and sibling (arrow) cells (inset, higher magnification). (C) A late stage 14 aMpT (Cut, magenta) stained for dpERK (green). dpERK is detected in the distal-most tubule cells, declining proximally towards the kink. High dpERK associated with the proximal tubule probably corresponds to hemocytes (asterisk) clustered around the MpTs [64]. Arrowhead, TC. A heat map showing dpERK is shown in (C′). (D) Stage 15 aMpT (Cut, magenta) in a *Capicua::Venus* (GFP, green) embryo. (D′) A heat map reveals high levels of nuclear Capicua in proximal tubule cells, while Capicua is absent or present at low levels in the cytoplasm of distal cells. Arrowhead, position of TC. (E) Quantification of dpERK activation along the D-P axis for ∼60 µm of the distal tubule (spanning ∼ten cell diameters) from the TC from stage 13 embryos. Average intensity (blue curve) of dpERK staining (*n* = 7 tubules) is plotted against tubule length (µm). Error bars (grey area) = standard error of the mean (SEM). Red line; best fit curve using fourth-order regression analysis. (F, F′) Stage 13 tubule stained for Cut (F) or dpERK (F′) showing tubule region measured in (E). D-P distance from TC in µm (red numbers), TC (arrowhead). (G, G′) Stage 16 *Df(os)1* embryo stained for Cut (white) showing that both anterior (aMpT) and posterior (pMpT) tubules lack TCs and SCs and fail to undergo normal elongation. (H, I) Embryos cultured to stage 16 following laser ablation of the TC and SC at late stage 12. Tubule cells, Cut (red); TCs, 22c10 (anti-Futsch, green). (H′, I′) Higher magnification views. TC ablated tubules fail to undergo elongation, whereas control tubules elongate normally. Distal (d) and proximal (p) tubule ends; arrowheads TC (I, I′) or ablated site (H, H′).

Previous work [26],[27] has shown that tubules cultured *in vitro* from stage 11 are able to elongate outside their normal environment, suggesting that tubule CE movements are regulated by mechanisms intrinsic to the tubule. Our previous work has also indicated that the distal-most cells in the tubule, the tip cell (TC) and its sibling (SC) (Figure 2A), are important for tubule elongation [12],[26],[28].

Both terminal cells secrete the EGF signal Spitz (Spi) during stage 12 to promote tubule cell proliferation [29]–[31]. However EGF signalling from the TC lineage persists beyond stage 12 throughout the period of tubule elongation, as revealed by the expression of the protease Rhomboid that cleaves Spi to produce its secreted and active form (sSpi) (Figure 2B). Staining for a read-out of signalling, diphosphosphorylated extracellular signal-regulated kinase (dpERK) [32], shows that the EGF pathway is activated in tubule cells through to stage 16 and it appears that activation is stronger in distal (close to the TC) compared to proximal tubule regions (Figure 2C and 2C′). Quantitative analysis of dpERK levels confirms graded activation in response to signalling, highest closest to the TC and declining towards the point of tightest tubule curvature (the kink) approximately half way along its D-P length (Figure 2E and 2F). These observations were confirmed using a second assay for pathway activation. Capicua is localised to the nuclei of quiescent cells but is translocated to the cytoplasm upon activation, where it is processed for degradation [33],[34]. Confirming our findings for di-phosphorylated ERK, expression of a tagged Capicua::Venus construct is diminished distally but remains high in the proximal tubule (Figure 2D and 2D′).

### EGF Signalling Is Required for Cell Intercalation and CE Movements in the Tubules

We ablated both the TC and SC either genetically or physically using a laser. In embryos mutant for the proneural genes [35] or components of the JAK/STAT pathway (BD, unpublished data), the TC lineage is not specified, tubules lack TCs and SCs and fail to undergo elongation (Figure 2G and 2G′). However the late phase of tubule cell proliferation also fails in the absence of the TC lineage [26],[35]. To test whether the reduced cell number contributes to elongation defects we laser ablated the TC and SC in late stage 12 embryos when tubule cell proliferation is complete. We used *ctB>UAS-CD8-GFP* to mark all tubule cells and centred our ablation on the distal three or four cells to ensure TC/SC removal. By stage 16, tubule elongation had failed completely in tubules lacking a TC and SC (Figure 2H and 2H′), whereas the contralateral (non-ablated) tubules underwent normal elongation (Figure 2I and 2I′). Together these experiments show that the TC lineage is required for tubule elongation.

**Figure 3:**
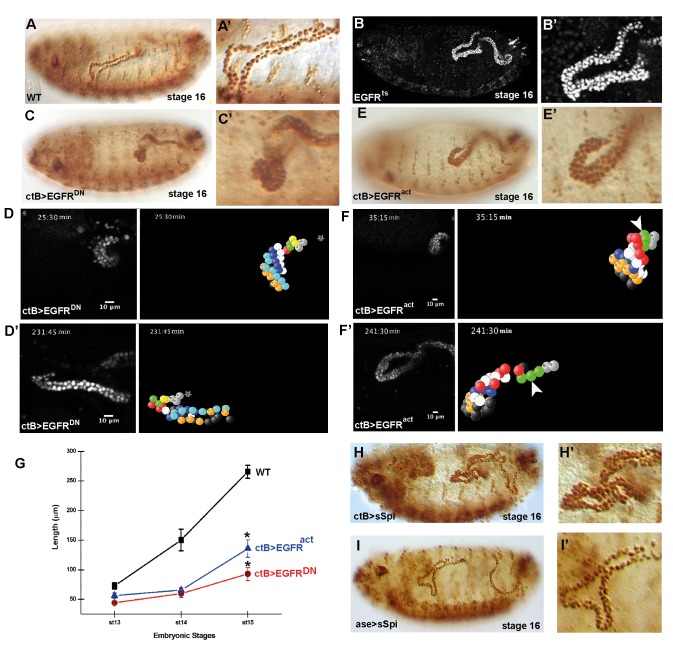
Polarised EGF signalling is required for MpT elongation. (A–C, E) Stage 16 embryos stained for MpTs (Cut). (A) Wild type embryo. (B) *EGFR^ts^* embryo raised at restrictive temperature during period of elongation. (C) *ctB>EGFR^DN^*, (E) *ctB>EGFR^act^*. Perturbation of EGF signalling disrupts tubule elongation. (D, D′, F, F′) Beginning and end point still images from Movies S6 and S8 in *ctB>EGFR^DN^* (D) and *ctB>EGFR^act^* (F) embryos, right panels show 4-D reconstruction, stars in (D, D′) indicate TC. Circumferential cell rows (coloured) achieve no (D,D′) or highly reduced (F,F′) cell intercalation (arrowheads). (G) Graph to show tubule elongation in wild type embryos and in those with perturbed EGFR signalling. Tubule length is significantly reduced in *ctB>EGFR^DN^* (*p* = 4×10^−6^<0.05) and *ctB>EGFR^act^* (*p* = 1×10^−4^<0.05) compared to WT. (H, I) Stage 16 *ctB>sSpi* (H) and *ase>sSpi* (I) embryos stained for Cut. (H′, I′) Enlarged views showing aMpTs.

To test the role of EGF signalling in tubule extension we abrogated signalling after the completion of EGF-dependent tubule cell division using the temperature sensitive allele of the epidermal growth factor receptor (*EGFR^f7^*) [29],[36]. In the majority of embryos shifted to the restrictive temperature at mid-stage 13, tubule elongation is severely disrupted (87%, *n* = 15 cf controls raised at the permissive temperature 5%, *n* = 18). The tubules remain short and the number of cells encircling the lumen fails to reduce as in sibling control embryos (Figure 3A and 3B). Similar defects in tubule elongation occur when EGF signalling is disrupted by expressing a dominant negative receptor [37] using the Gal4 driver *ctB*, which represses signalling only after all tubule cell divisions have ceased (Figure 3C, 3D, and 3G; Movies S5 and S6) [31]. The analysis of tracked cells from movies of tubules expressing the dominant negative receptor reveals that very few cells complete intercalation in contrast to the wild type (Figure 1H; Movies S2 and S4). Cell movement is strongly reduced; the average speed of movement is 0.5±0.07 µm min^−1^ *n* = 42 distal cells (cf wild type intercalating cells 1.14±0.02 µm min^−1^).

Surprisingly, experiments in which EGF pathway signalling is hyperactivated in all tubule cells, either by expressing the constitutively activated receptor λTop/EGFR^act^ [38] or active ligand sSpi [39], also produce striking defects in tubule extension, strongly reminiscent of the loss of function phenotypes; 64% (*n* = 28) of tubules expressing the activated receptor fail to elongate, remaining short and thick (Figure 3E–3H; Movies S7 and S8). Tracking cells in activated tubules shows that, as in the loss of EGF pathway function, the number of cell intercalations is reduced, although those in the most distal region close to the source of ligand still occur at near the wild type rate with intercalations taking 37±4.3 min, *n* = 10 distal cells (Figure 3F and 3F′; Movies S7 and S8). However overall cell movement is much reduced (0.6±0.06 µm min^−1^, *n* = 38 cells cf wild type 1.14±0.02 µm min^−1^). In contrast, enhanced expression of sSpi from the TC lineage, which is likely to induce higher than normal levels of EGF pathway activity whilst retaining its spatial asymmetry, does not lead to defective tubule elongation (Figure 3I).

Together these experiments show that the TC lineage is the source of the EGF ligand, Spitz. They also reveal that asymmetric, signalling from a localised source is crucial for tubule elongation, as either loss of receptor activation or hyperactivation along the whole tubule length disrupts CE movements.

### Tubule Cells Are Polarised within the Plane of the Epithelium and This Polarity Depends on EGF Signalling

**Figure 4:**
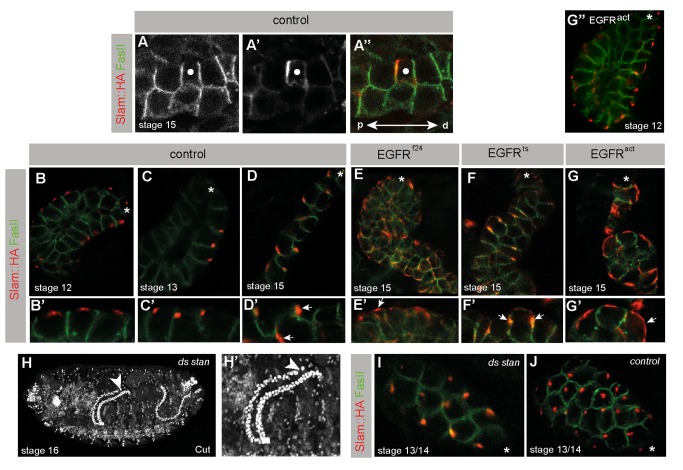
EGFR signalling controls planar polarity in MpT cells. (A–G) MpTs expressing Slam-HA (red) stained for FasII (green). (A–A″) Single cell clone (white dot) expressing Slam-HA in a stage 15 tubule. Slam-HA is highly enriched only at the proximal cell membrane. p-d, proximo-distal tubule axis. (B–D) Timeline of Slam-HA accumulation in wild type MpT cells. (B) Stage 12, Slam-HA localises centrally in small single puncta on the basal membrane. (C) Stage 13, basal puncta shift proximally. (D) By Stage 15 Slam-HA has spread along the proximal cell membrane. (B′–D′) higher magnification views. Arrows Slam-HA. (E–G) Perturbation of EGF signalling disrupts Slam-HA localisation from late stage 13 (G″) shows Slam-HA localised normally to the basal cortex of stage 12 *EGFR^act^* tubule cells). (E) Stage 15 *EGFR^f24^*, F *EGFR^ts^* (raised at the restrictive temperature from stage 13) and (G) *EGFR^act^*, tubule cells show randomised membrane distribution of Slam-HA. (E′–G′) higher magnification views, arrows Slam-HA. (H) Stage 16 *ds,stan* double mutant embryo shows normal tubule elongation (Cut, white). Arrowead, TC. (I, J) Stage 13/14 *ds,stan* (I) and control (J) MpTs expressing Slam-HA (FasII, green). Slam-HA is distributed normally in *ds,stan* mutant tubules. Asterisk in (B–G, I, J); distal tubule tip.

These data suggest the idea that the Spitz signal establishes an axis of polarity along the tubule length, about which directed cell rearrangements, required for orderly cell intercalation, occur. We therefore asked if tubule cells exhibit planar polarised features, which are dependent on EGF signalling. As tubule cells lack visible polarity landmarks we relied on a technique that has revealed polarity in other tissues: the expression of the membrane-associated Slam protein. During germ band extension Slam assumes a bipolar distribution on the vertical cell membranes orthogonal to the A-P axis that presages cell intercalation [6],[13]. Expression of a tagged form of Slam (Slam-HA [40]) in tubule cells (Figure 4A–4D) reveals that it becomes planar polarised at the onset of CE. In wild type tubules prior to CE (before stage 13) Slam accumulates in a single central clump in the basal cortex of each cell (Figure 4B). At stage 13 (Figure 4C), Slam relocates towards the basal proximal cortex. The proximal localisation is maintained throughout the period of intercalation during stages 14 and 15 (Figure 4A and 4D) where it appears to spread down the lateral cell membrane. To show conclusively that Slam localises preferentially to the proximal cortex we induced Slam expression in single cells (Figure 4A) where it accumulates on the proximal side and is virtually absent from the distal cortex (Figure 4A and 4A″).

These data provide the first evidence that tubule cells are polarised within the plane of the epithelium and reveal that aspects of planar polarity are established before the initiation of tubule extension. Slam is not normally expressed in the tubules [41]. Thus while Slam localisation reveals latent polarity in the tissue, it does not contribute to the mechanism by which the tissue is normally polarised.

Using ectopic Slam as a marker for tissue planar polarity, we assessed tissue polarity in tubules in which EGF signalling was perturbed. Before stage 13, Slam-HA localisation is unaltered in conditions of either loss- or gain-of-function EGF signalling; Slam is found in a central cluster in the basal cortex of tubule cells (Figure 4G″). However, Slam fails to redistribute to the proximal cortex and becomes severely disorganised as development proceeds. At the time when Slam would normally relocate proximally, it either fails to relocate or spreads around the entire cell (Figure 4E–4G). These results show that in the absence of EGF signalling, or under conditions of global pathway activation, tubule cells are unable to polarise within the plane of the tissue, supporting the hypothesis that EGF signalling is the source of vectorial information that establishes and/or maintains planar polarity in the tubule.

Other studies have shown that EGF signalling is required for the maintenance of apicobasal cell polarity and also for cell survival [42]. Defects in either could account for defective tubule development and so we analysed these parameters after perturbing EGF signalling in tubules. Neither driving a dominant negative EGF receptor nor activating the pathway in all tubule cells alters their apicobasal polarity, as revealed by the distribution of the apical marker Bazooka and the lateral membrane protein FasII (Figure S1A–S1C). There is no cell death in control tubules during stage 15 detected by cleaved Caspase 3 staining (Figure S1D, see S1G and S1H for positive control), and we find no increase in cell death in the tubules either when the EGF pathway is abrogated or hyperactivated (Figure S1E and S1F). These data indicate that the primary response of tubule cells to asymmetric and graded EGF signalling is the acquisition of PCP.

### PCP Gene Activity Is Not Required for Tubule Elongation

The so-called PCP genes play key roles in regulating tissue polarity in diverse organisms [43],[44]. Furthermore, PCP signalling has been implicated in some [21],[22],[24], but not all [13], tissues undergoing CE movements. We therefore asked whether the PCP genes contribute to tubule planar polarity and elongation. There is evidence for two independently acting PCP systems, the Dachsous (Ds) and Starry night (Stan) systems [45]. For this reason we examined mutations in genes for each system independently (removing maternal and zygotic contributions) and double mutant combinations that remove the function of both systems together (see Table S1 for the lines examined). No defects in tubule extension were found for any of the mutants or double mutants we tested (Figure 4H and 4H′; Table S1), indicating that the PCP genes are not required for CE movements in the tubules. Furthermore, we found that Slam localises normally in the absence of PCP gene function (Figure 4I and 4J), revealing that the PCP genes we have tested are also dispensable for planar polarity in the developing tubule.

### Cytoskeletal Dynamics in Tubule Cells Are Regulated by EGF Signalling

**Figure 5:**
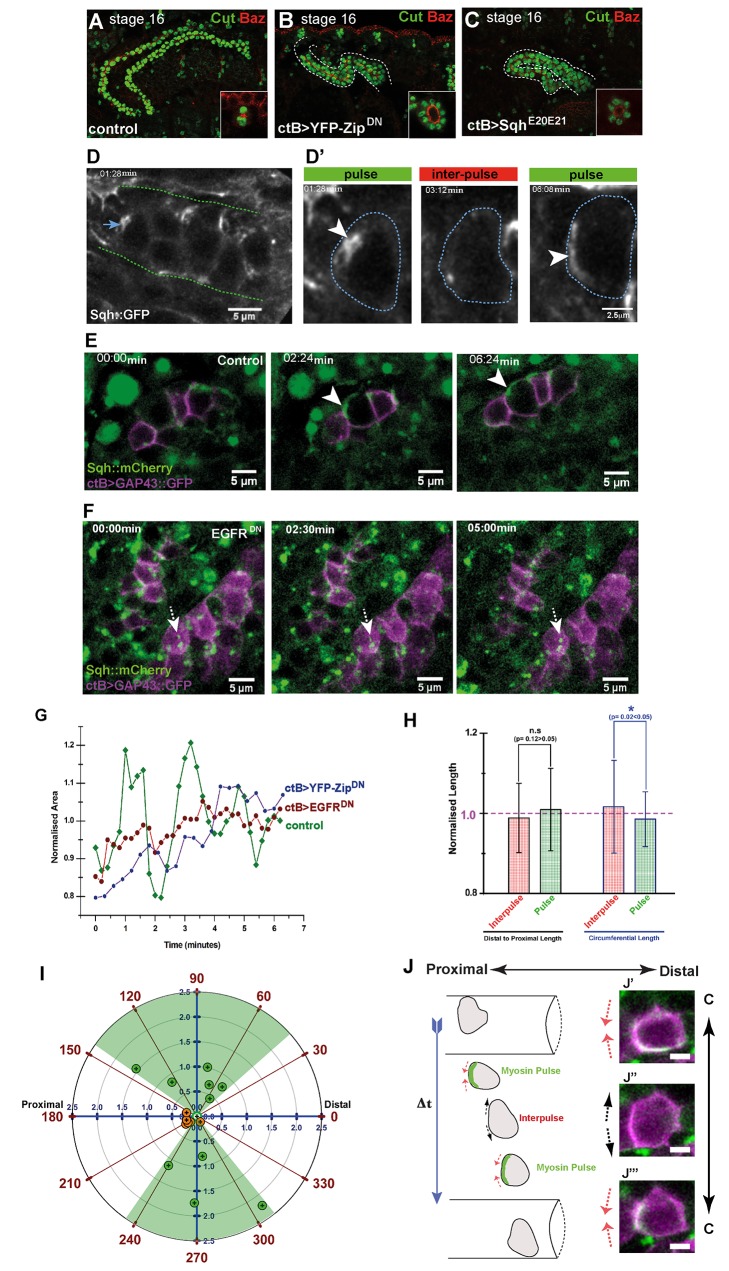
Polarised basal Myosin II pulses drive circumferential movement of MpT cells. (A–C) Stage 16 MpTs stained for Cut (green) and Baz (red) in control (A), *ctB>UAS-YFP-Zip^DN^* (B), or *ctB>UAS-Sqh^E20E21^* (C) embryos. Insets in (A–C) show cross-sections through tubules. (D) Basal view of ∼ten cells in the distal tubule region (dashed lines), showing Myosin II/Sqh::GFP (white) from Movie S9. Distal is to the right. (D′) Time-lapse images of the single cell arrowed in (D) (blue dashed lines) showing Myosin II (arrowhead) accumulating in dynamic pulses localised asymmetrically to the proximal cortex. Inter-pulse periods lack discernable Sqh::GFP accumulation (Movie S10). (E, F) Still images from control (E, Movie S11) or *EGFR^DN^* (F) (Movie S12) tubules (Myosin II/Sqh::mCherry, green; cell membranes, magenta). Proximal Myosin II pulses are absent in *EGFR^DN^* tubules, only static puncta of Myosin II are seen (F, dashed arrow). (G) Basal area of a control (green), *EGFR^DN^* (red) and YFP-*Zip^DN^* (blue) MpT cell over time. Basal area and circumferential length fluctuate widely in control compared to *EGFR^DN^* and YFP-*Zip^DN^* cells. 1.0 represents normalised average area. (H) Histogram of the coordinates of MpT cell shape in relation to Myosin II pulses. Normalised length of D-P and circumferential cell axes (*n* = 15 pulses and 25 interpulses in 10 aMpT cells). While D-P length shows no significant difference between pulse (green) and interpulse (red) periods (*p* = 0.12>0.05), circumferential length decreases significantly during pulses (*p* = 0.02<0.05). 1.0 (dashed line) is the normalised average D-P or circumferential length; error bars ± standard deviation (SD). (I) Polar plot showing displacement of single cell centroids in control (green circles, *n* = 10 cells) and *EGFR^DN^* (orange circles, *n* = 6 cells) MpTs. Tubule D-P axis 0°–180°, circumferential axis 90°–270°. Radial rings indicate speed of cell movement (µm min^−1^) and central open circle marks the starting position for all cells. Control cell movement is biased along the circumferential axis (shaded green). *EGFR^DN^* cells move very little and their final position remains aligned with the D-P axis. (J) Cartoon of cell-shape changes and movement caused by the proximal Myosin II pulse (green shading). Oscillating circumferential cell length (red and black arrows) correlates with myosin pulses, allowing cells to inch around the tubule circumference. Tubule outline and cell position over time is shown. Panels on the right show basal views of a cell labelled for membrane (GAP43::GFP, magenta) and Myosin II (Sqh::mCherry, green). Change in circumferential cell length (C, double headed arrow) during proximal Myosin II accumulation (white, J′–J′″) can be seen. Scale bar = 3 µm. See also Figure S4B and Movie S14.

Previous studies have shown that the normal activity of non-muscle Myosin II is required for tubule elongation [46],[47]. However the phenotypes reported were quite weak perhaps because both Zipper (Zip, Myosin heavy chain) and spaghetti squash (Sqh, myosin light chain) are supplied maternally (http://insitu.fruitfly.org/cgibin/ex/report.pl?ftype=1&ftext=CG15792). We therefore assessed the effects of perturbing Myosin II activity in tubule cells by driving the expression either of a dominant negative Zipper (YFP-Zip^DN^) (Figure 5B) [48] or of constitutively active Sqh (*Sqh^E20E21^*), which is known to result in an increase in Myosin II activity (Figure 5C) [49],[50]. Compared to wild type tubules, those with altered Myosin II activity fail to extend normally but remain short with more than two cells around the circumference of the lumen (Figure 5A–5C and 5G, Movie S13; YFP-Zip^DN^ 95% and *Sqh^E20E21^* 75% of embryos showed elongation defects by stage 15 [*n* = 20 in each case]). These data show that normal levels of Myosin II activity are required for cell intercalation and tubule elongation.

Expression of a tagged Myosin II light chain, Sqh::GFP, in a *sqh* mutant background allowed us to analyse the activity of myosin in tubule cells during elongation. This construct rescues the embryonic *sqh* mutant phenotype [6],[49] indicating that endogenous levels of expression are maintained. Movies of the basal-most side of cells during tubule extension reveal regular pulses of Myosin II activity, in which cytoplasmic spots of myosin move to the proximal cortex of cells (Figure 5D; Movies S9 and S10). Analysis of 63 pulses in 37 tubule cells from eight different embryos indicates that the duration of pulses ranges from 0.9 to 4.1 min (average 1.98±0.08 min) with 0.13 to 3.6 min (average 1.78±0.2 min) between pulses (interpulse). Of 61 pulses analysed, 49 showed basal, proximal enrichment and only 12 showed proximal-to-medial or proximal-to-distal movement of myosin. It is striking that although spots of myosin fluorescence can be seen at the orthogonal, circumferential cortices, enriched crescents are almost never seen in these regions.

In order to follow the localisation of Myosin II more precisely we double labelled tubule cells with Sqh::mCherry [51] and GAP43::GFP to label cell membranes. Movies show dynamics identical to those using Sqh::GFP (Figure 5E; Movie S11). When driving a dominant negative EGFR construct, where cells fail to intercalate and tubule extension is lost (Figure 3C, 3D, and 3G), the pulses of active myosin completely fail (Figure 5F; Movie S12).

Actomyosin dynamics are associated in other systems with alterations in cell shape, cell movement, and rearrangement [6],[51]–[53]. We analysed fluctuations in the basal shape of tubule cells by measuring their area, D-P, and circumferential lengths. This analysis reveals that cell shape is in a constant state of flux (Figures 5G, S2, and S3A-S3C). We compared the dynamics of fluctuations during a Myosin II pulse with interpulse periods in individual cells but found no obvious correlation with cell shape (Figure S2). However, averaging measurements from multiple cells (*n* = 10) revealed that while pulses caused no significant change in the D-P axis, there is a small but significant decrease in circumferential length associated with Myosin II pulses (Figure 5H).

In contrast, driving the expression of YFP-Zip^DN^ in tubules results in a strong reduction in cell dynamics with much reduced fluctuations in cell area (Figures 5G and S3A″) and little change in either the circumferential or D-P axial length of cells (cf shaded areas in Figure S3B, S3C, with S3B″ and S3C″). The dynamic fluctuations in cell shape are also dramatically reduced when EGF signalling is compromised; in tubules expressing EGFR^DN^ the basal area of cells (Figures 5G and S3A′) and their axial lengths (Figure S2B′ and S2C′) scarcely alter compared with the fluctuations seen in wild type tubule cells (Figure S2A–S2C). Tracking cell trajectory over time (Figure 5I) shows that control cells move in a circumferential direction (± approximately 50°). In tubules expressing either EGFR^DN^ or YFP-Zip^DN^ the small cell movements that occur fail to show any bias towards the circumferential axis (Figures 5I and S3D).

Together our analysis of cell behavour in control and mutant tubules in which EGF signalling is deranged or where the normal activity of Myosin II is compromised indicates that proximally directed pulses of cortical Myosin II are essential for cell intercalation but occur only in cells that have been planar polarised by asymmetric EGF signalling. In control tubule cells these pulses produce a transient, small but significant reduction in the circumferential length of cells enabling them to move in this axis (Figure 5J), resulting in the cell rearrangements that produce tubule elongation. Without polarised EGF signalling the pulses fail, cells dynamics are dampened and intercalation is either much reduced (pathway activation) or fails (loss of the pathway activity) so that tubule elongation is compromised.

### The Processes Underlying Elongation of the Proximal Half of Tubules Differ from the Distal Half

Our analysis of the response to EGF signalling in tubules has established graded activity but only in the distal half of the tubuels (Figure 2E and 2F). We therefore wondered whether cells in the proximal part of tubules become planar polarised. Expression of Slam-HA in tubules from stage 13–16 embryos reveals that the protein in the proximal tubule, in contrast to the distal half, is not asymmetrically distributed at any stage during elongation (stage 15 shown in Figure S4A and S4A″). As we have correlated the acquisition of planar polarity in cells with the development of dynamic, asymmetric subcellular activity of Myosin II, we wondered whether cells in the proximal region exhibit similar cytoskeletal dynamics. Imaging tubules expressing Sqh::mCherry (Figure S4B and S4B″; Movies S14 and 15) reveals that in contrast to distal regions, where repeated, proximally localised crescents of Myosin form, there is no apparent asymmetric Myosin activty in the proximal half of the tubules.

These data suggest that the mechanism by which cells in the proximal half of the tubules intercalate differs radically from the distal half and might not depend on polarised cytoskeletal activity, resulting in circumferential cell movements.

### Tubule Morphogenesis Underpins Tissue Physiology and Homeostasis

**Figure 6:**
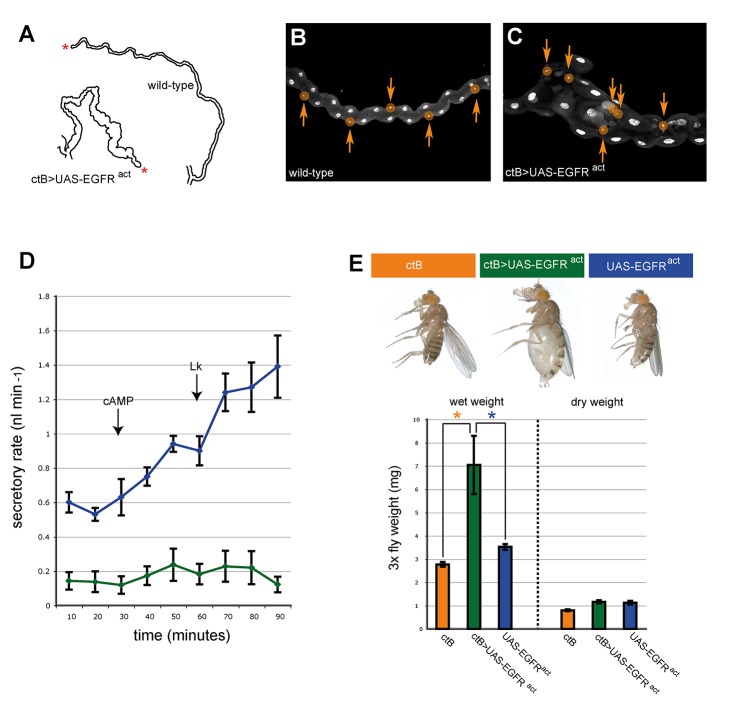
Normal tubule elongation is important for renal function. (**A**) Representative traces of wild-type (upper) and *ctB>EGFR^act^* (lower) 3rd instar tubules. Asterisk, distal tubule tip. (**B**, **C**) 3rd instar distal tubule regions (DAPI, nuclei; stellate cells, smaller nuclei coloured orange). The regular spacing of stellate cells (arrows) is disturbed when EGF signalling is activated in all tubule cells. (**D**) Secretory rates (nl min^−1^) of control (blue) and *EGFR^act^* (green) MpTs. Cyclic adenosine monophosphate (cAMP) and Leucokinin (LK) were added at 30 and 60 minutes, respectively (arrows). *EGFR^act^* tubules have strongly reduced basal secretory rates and are refractory to diuretic stimulation. (**E**) Adult flies aged for ∼24 hours after eclosion. *EGFR^act^* animals are grossly bloated compared with controls. Wet and dry weight measurements from *ctB-Gal4* control (orange, *n* = 5), *ctB>EGFR^act^* (green, *n* = 11) and *UAS-EGFR^act^* control (blue, *n* = 13) animals. Weight (mg) for the weight of three flies is given on y-axis. Error bars = standard deviation (SD). The difference in wet weight between control and experimental animals is highly significant, *p* = 2.82×10^−6^ (orange asterisk) and *p* = 8.66×10^−10^ (blue asterisk). Dry weights are approximately equal.

MpTs are the major organ for excretion, ionic balance, and osmoregulation in the majority of insects [9],[10]. Toxins are cleared from the haemolymph by active transport and primary urine is secreted into the tubule lumen by two cell types in the distally placed transitional and main segments. Homeostasis is accomplished by modification of primary urine as it passes down more proximal regions of the tubule before emptying into the hindgut via the ureters. We asked whether the final tubule shape is important for its function. Embryonic tubules persist through larval life and metamorphosis and so are retained in the adult. As there are some escapers when *ctBGal4* is used to drive the activated EGF receptor we examined tubules from adult flies of this genotype. Their tubules are abnormal in shape compared to controls, being shorter and wider with conspicuous bulges (Figure 6A). This shows that embryonic tubule defects are not rectified during later developmental stages. In the distal two-thirds of wild type tubules stellate cells, responsible for anion and water movement in the secretion of primary urine, are regularly interspersed with the cation-transporting principal cells (PCs) (Figure 6B). Stellate cells are present in the abnormally shaped *ctB>UAS-EGFR^act^* tubules but their regular spacing is severely disrupted (Figure 6C).

Defects in tubule shape and in the organisation of specialised secretory cell types could well compromise renal physiology. To test this possibility, we compared tubule secretion in control and *ctB>UAS-EGFR^act^* tubules using an established *in vitro* tubule secretion assay [54],[55]. In control tubules the unstimulated, basal rate of primary urine secretion was 0.59 nl min^−1^ (*n* = 10). In contrast 4/11 *ctB>UAS-EGFR^act^* tubules did not secrete at all. The average basal rate of secretion for the remaining *ctB>UAS-EGFR^act^* tubules was 0.2 nl min^−1^ (*n* = 7). After stimulation with the diuretic activators cAMP and Leukokinin (LK) control tubules increased their secretory rate to 1.39 nl min^−1^ while *ctB>UAS-EGFR^act^* tubules failed to show any increase in secretory rate (Figure 6D). These data clearly demonstrate that secretory rate, a direct measure of tubule function, is either abolished or significantly reduced when tubule morphogenesis is disrupted.

The impact of defective tubule elongation on the physiology of adults can be seen within 24 hours of eclosion. Compared with control animals, *ctB>UAS-EGFR^act^* adults have grossly distended abdomens and mouthparts (Figure 6E), indicative of fluid retention through defective osmoregulation. We confirmed that distention was due to fluid retention (and not gas) firstly by pricking submerged flies, which led to abdominal deflation without gas bubbles. Secondly, we compared wet weight versus dry weight in experimental and control flies. *ctB>UAS-EGFR^act^* adults are over twice as heavy as control flies when measured wet, while dry weight measurements are not significantly different (Figure 6E; data for females, equivalent results were obtained for males). These data indicate that osmoregulation is severely compromised in *ctB>UAS-EGFR^act^* adults. Together our data illustrate the critical importance of tubule shape both for effective physiological function of the organ system and homeostasis in the whole animal.

## Discussion

**Figure 7:**
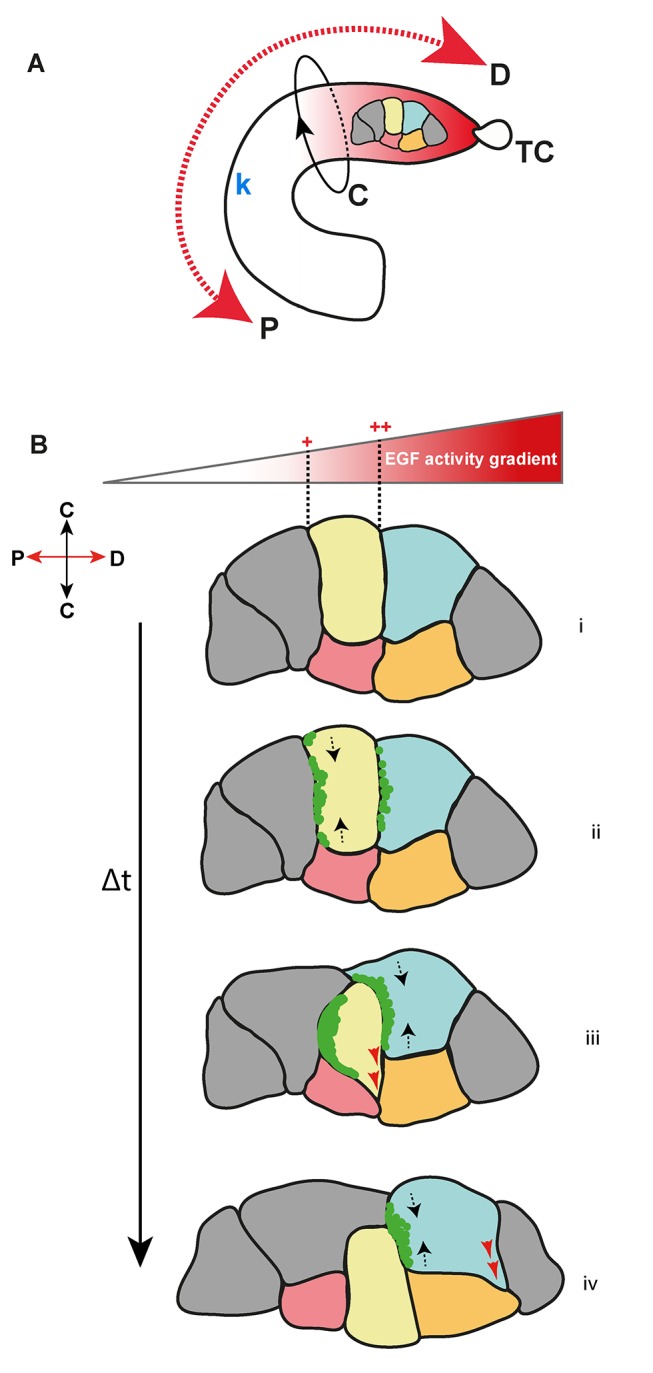
A model for MpT cell intercalation. (A) Schematic showing a stage 13 MpT with distal to proximal (D–P) and circumferential (C) coordinates indicated. An asymmetric source of EGF ligand from the distally placed TC (TC) establishes a gradient of EGF pathway activity (red shading) in the distal tubule extending to the kink (k). A small cluster of tubule cells is highlighted. (B) Basal view of this cluster of cells during elongation. Individual cells read differential EGF activity across their D-P axis (higher distal relative to proximal; dashed lines in i), to produce an asymmetric accumulation of Myosin II (green, ii) at the basal, proximal cortex (buff cell). This leads to contraction along the circumferential axis (dashed arrows, ii). The resulting change in cell shape facilitates progressive, small movements (red arrows) between circumferential neighbours (pink and orange cells, iii). Multiple cycles of Myosin II pulses lead to cell intercalation (iv). Asynchronous pulses in a neighbouring cell (cyan), contracts its circumferential axis (black arrows, iii) facilitating intercalation of the buff cell, and producing a change in cell shape to initiate another cell intercalation event between the orange and grey cells (red arrows).

In many situations tissue morphogenesis results from orderly cell rearrangements, which require the integration of positional information and oriented cell intercalation. Our data show that in fly renal tubules axial information is provided by an asymmetric EGF signal from a localised source, which acts to polarise cells in the distal half of the tubule just as elongation is about to start. The acquisition of D-P PCP leads to asymmetric, proximally directed pulses of myosin, which in turn result in repeated small contractions of the cell in the circumferential axis. Over time this results in the intercalation of cells around the tubule circumference to produce tubule elongation. While previous reports in other systems have focussed on parts of this sequence of events [6],[7],[13],[21],[56],[57], we have identified the source of polarisation, established the axis of planar polarity at the cellular level, and shown that it is required for the asymmetric behaviour of Myosin II motors that ensure oriented cell rearrangements (Figure 7).

Polarity in the plane of an epithelium is frequently conferred by the activity of PCP genes [43],[44],[58], and in many cases CE movements depend on the expression of these genes [24]. However the patterning of cell movements during tissue morphogenesis or collective migration have also been shown to require the activity of other pathways. In *Drosophila* the extension of the germ band depends on the expression of the early patterning pair rule genes [8],[13], *Drosophila* hindgut elongation depends on JAK-STAT pathway activity [56], and border cell migration in the egg chamber is polarised by gradients of receptor tyrosine kinase (RTK) signalling [59],[60]. The sensitivity of border cells to differences in ligand levels across a single cell diameter is enhanced by spatially regulated receptor endocytosis and processing. This depends on Cbl (a RTK-associated E3 ubiqitin ligase) and Sprint (a pathway activated Rab5GEF), which together act to down-regulate RTK receptors asymmetrically, leading to enhanced levels of pathway activation at the leading (higher ligand) cell face [61]. We find that both Cbl and Sprint are required for normal CE movements during renal tubule elongation [62],[63], showing that the enhancement of polarised RTK activation also occurs in this tissue.

The gradient of pathway activation is clear in the distal half of the tubules during elongation, as revealed by dpERK staining or Capicua::Venus expression and quantification suggests that the graded response extends over a considerable distance, approximately 60 µm (ten cell diameters). Such a comparatively long-range effect could result from the secretion of ligand from a localised source, particularly if diffusion of ligand into the haemolymph were restricted either by the extracellular matrix, known to ensheath the tubules [64], or by secretion of ligand through an apical route into the tubule lumen. The ultrastructure of the TC is consistent with apical secretion ([26] and HS, unpublished data), and the distribution of Rhomboid, which is enriched apically in both TCs and SCs, favours this hypothesis (Figure 2B) [31]. Alternatively, short-range signalling from the TC lineage might act to break axial symmetry, followed by local interactions between cells to propogate polarity. We hoped that it would be possible to distinguish between these models by generating clones of cells with altered EGFR activity or ectopic ligand secretion in order to assess non-cell autonomous effects of altered signalling on cell polarity. However clones generated even at syncytial stages of embryogenesis yield tubule clones that do not exceed two to three suitably labelled cells, ruling out the validity of this approach (see Figure S5).

Our analysis also revealed that there is little discernible expression of dpERK or modulation of Capicua::Venus in the proximal half of the tubules and this is reflected in the lack of any polarisation in the distribution of cortical Slam-HA or of asymmetric actomyosin activity. These findings indicate that, although tubule extension in the proximal regions, as in the distal, results from circumferential intercalation of cells, underlying movements must be regulated by different processes in the two halves. Our analysis has focussed on the anterior tubule pair, whose forward movement through the body cavity is regulated by guidance cues expressed by specific target tissues [64], and we suggest that extension of the distal tubule results in sufficient forward movement to deliver the cue-responsive kink region close to target tissues that promote continued forward movement of the whole tubule. As the tubule is tethered both to the ureter/hingut proximally and distally, by TCs/alary muscle contacts [65], forward tubule movement will produce mechanical forces that could promote circumferential intercalation in the proximal tubule half.

In the distal tubule, one important consequence of cell polarisation is asymmetry in the activity of Myosin II to the proximal side of tubule cells, which leads to transient circumferential cell contraction. Oriented Myosin II accumulation has been shown to result from EGF signalling in the tracheal placode; in the absence of signalling, Myosin II accumulation remains punctate and dispersed [66]. We find a similar phenotype. When EGF signalling is perturbed, Slam fails to become localised and Myosin II remains dispersed in unpolarised cells so that pulses fail altogether.

A novel observation of this study is that planar Myosin II pulses are required in the basal cortex of MpT cells for CE. This contrasts with findings in the extending germ-band where adherens junction remodelling resulting from apical planar actomyosin enrichment has been proposed as a major motive force for cell rearrangements [6],[7],[15]. In MpT cells, asymmetric Myosin II activity within 4 µm of the basal surface correlates with cell shape change and is required for CE movements. We have examined the apical side of tubule cells for actomyosin dynamics and do not detect repeated or polarised Myosin II cresents in this region of the cell cortex (see Movie S15), but we have not been able to live image the apical regions deeper in the tubules with sufficient reliability to assess whether junctional remodelling precedes or follows the basal changes. He and colleagues [53] have shown that oocyte elongation depends on pulsatile basal contractions of follicle cells, oriented in the circumferential axis. Similarly elongation of the *Caenorhabditis elegans* embryo results from the intercalation of hypodermal cells led by basal, medially directed protrusions [67]. It is possible that intercalating cells commonly initiate movements basally and that in epithelia junctional remodelling follows, also contributing actively to tissue morphogenesis [24].

A remaining question is how the acquisition of PCP relates to asymmetry in cytoskeletal activity. Slam localisation during cellularisation of the *Drosophila* embryo or in the extending germ band is known to highlight sites of Myosin II accumulation [6],[13],[68]. Like the extending germ band, tubule cells do not express Slam endogenously; its localisation therefore must reflect asymmetry in a binding partner, such as RhoGEF2, to which it is known to bind during cellularisation [68]. An antibody against RhoGEF2 revealed that its expression is scarcely detectable in tubule cells and it does not appear to be asymmetrically localised. However, mutants for *RhoGEF2* show CE defects in tubule elongation [69]. This suggests that RhoGEF2 might provide a link between EGF signalling, tubule cell polarity and asymmetric cytoskeletal activity.

The duration of myosin pulses and cell circumferential contraction is approximately 2 min, while cell intercalation takes an average of 42 min. During tubule elongation the diameter of cells is around 5 µm (see Figure 5) yet our measurements indicate that cells move an average of 1 µm min^−1^. Are these measurements consistent with the dynamics and timing of tubule morphogenesis as a whole? If one assumes that cells move in a consistent direction during intercalation this would suggest a serious overshoot so that cells would move past their neighbours. However cell movement relative to neighbouring cells is not uniform (see Movie S4) and the movements we measure result from multiple factors; (a) the displacement of the whole tubule as a result of gut morphogenesis (for example; hindgut elongation during stages 13–16 [70]); (b) the concerted movement of tubule cells as a result of cell rearrangements in more distal regions; and finally (c) movements of individual cells relative to their neighbours that produce cell intercalation. The first two tend to produce distal-to-proximal movement, which would explain the deviation in total cell movement from the circumferential axis seen in Figure 5I. Our observations concerning circumferential movement suggest that cell intercalation results from repeated, transient, and very tiny movements (Figure 7iii)—which must be stabilised perhaps by adhesion either to the basement membrane or to adjacent cells—in which a small but consistent circumferential bias eventually achieves cell rearrangement (Figure 7iv).

Concerning timing; tubules increase in length 4-fold with the reduction of eight to 12 cells around the lumen to just two cells (Figure 1A, 1C, and 1D). Simple calculation suggests that this would require that every cell intercalates twice, with some undergoing a third cell rearrangment. Tubule extension takes approximately 5 hours and each intercalation an average of 42 min, indicating that three intercalation events could be accommodated in the time-frame of elongation.

The mechanisms known to drive tubule elongation include oriented cell division and polarised changes in cell shape, as well as CE cell rearrangements [16],[19],[22],[71]. Here we show that fly renal tubules elongate predominantly by cell intercalation. In frog and mouse embryos renal tubule extension also depends primarily on cell rearrangements as the orientation of cell division is random [16],[22]. As in *Drosophila* CE depends on Myosin II activity that is polarised in the plane of the tubule epithelium to bring about mediolateral cell intercalation. But in contrast to our findings, nephron elongation results not from cell intercalation directed by graded EGF signalling but from PCP gene-regulated formation and resolution of multicellular rosettes [22]. However, EGF signalling does play an important role during mouse embryonic kidney development in regulating both nephron cell proliferation and morphogenesis and in collecting duct extension [72],[73].

The mature shape of tubular epithelial tissues is critical for their effective function. Abnormalities in the morphogenesis of renal tubules in mammals, for example in cystic kidney disease, results in defective excretory physiology leading to premature death [1],[24],[74]. Flies lacking the normal polarising signals that regulate renal tubule morphogenesis similarly suffer renal malfunction leading to lethality [75]. The identification of a genetically manipulable system in which to study the molecular interactions that lead from cell polarity to asymmetric cytoskeletal regulation, polarised cell movement and tissue shaping provides a powerful model for future analysis of tubule morphogenesis in health and disease.

## Materials and Methods

### Fly Husbandry

Flies were cultured on standard media at 18°C or 25°C with ectopic expression at 29°C. Embryos were collected overnight at 25°C (29°C for dual colour imaging) on apple-juice agar plates with yeast paste. The following stocks were used: Oregon-Red (wild type); *ctB*-*Gal4*; *UAS-RedStinger6*; *UAS-(EGFP)Stinger2*; *Capicua::Venus* (gift of E. Wieschaus); *UAS-EGFR^DN^* (gift of M. Freeman); *UAS-λtop^4.2/4.4^* (*UAS-EGFR^act^*, gift of T. Shupbach); *EGFR^f7^*; *UAS-Sqh^E20E21^*; *UAS-YFP-Zip^DN^* and *sqh^AX3^*, *sqh*-*Sqh::GFP*, *sqh*-*Sqh::GFP* (gift of T. Lecuit); *sqh-Sqh::mCherry* [51] (gift of A. Martin); *UAS-GAP43::GFP; sqh^AX3^*, *sqh*-*Sqh::GFP*, *sqh*-*GAP43::mCherry* [76] (gift of B. Sanson); *A37-LacZ* (*nrmLacZ*); *Df(os)1A*; *UAS-Slam::HA* [40] (gift of J. Zallen); *hs-flp122* (gift of J. Castelli-Gair Hombría), *tub>stop>Gal4* (gift of M. Landgraf), *FRT ds^UA071^*; *ds^UAO71^*, *stan^E5^*; *ds^UAO71^*, *stan^3^*; *dsh^1^*; *fz^1^*; *stbm^6^*; *ft^G-rv^* (gifts of J. Casal and D. Strutt).

### Generation of SLAM::HA Clones

*hs-flp122; tub>stop>Gal4/UAS-Slam::HA*; embryos were collected for 2 hours at 25°C and heat-shocked in a 37°C water bath for 10 minutes. Embryos were aged to stage 15 at 25°C, fixed and processed for antibody staining.

### Time-Lapse Imaging

Embryos were dechorionated in 50% bleach, washed extensively with double-distilled water, and oriented dorso-laterally to visualise anterior Malpighian tubules (aMpTs). Oriented embryos were mounted on type-1 coverslips with an evenly spread layer of glue (3M Scotch tape glue-Heptane). Care was taken not to compress the embryos. Mounted embryos were covered with Voltalef-3S or Halocarbon-10S oil. For dual colour imaging of Myosin II and membrane dynamics, embryos of the following genotypes were used: *w^−^*;*ctB*>UAS-GAP43::GFP, *sqh*-Sqh::mCherry/*sqh*-Sqh::mCherry;+; *sqh^AX3^*;*sqh-*Sqh::GFP;*sqh-*GAP43::mCherry; *w^−^;ctB*>UAS-GAP43::GFP, *sqh*-Sqh::mCherry/UAS-EGFR^DN^;*sqh*-Sqh::mCherry/+.

Images were acquired on Leica SP5 or Olympus FV1000 confocal microscopes with 488 nm and 561 nm lasers. An Argon ion laser was used for imaging GFP; dsRed and mCherry were imaged with a 561 nm diode laser. z-Stacks were acquired every 45 seconds for 5–6 hours with a water immersion 20×/0.7 NA objective to capture aMpT elongation (Figure 1C, 1F, 3D, and 3F). 60×/1.4 NA (Figure 5A) or 63×/1.4 NA (Figure 5B and 5C). Oil immersion objectives were used to visualise Myosin II and membrane dynamics in the basal-most 2–4 µm z-sections every 8–15 sec. Dual colour imaging was performed using previously established excitation band-pass settings [51] with Leica-SP5 Hybrid detectors. All images except those in Figure 5A were acquired on a Leica-SP5 confocal microscope. For Sqh::GFP in Figure 5A an Olympus-FV1000 confocal was used. All embryos completed development and hatched as L1 post-imaging.

### Analysis of Time-Lapse Sequences

aMpT lengths and cell shape changes were analysed using ImageJ (http://imagej.nih.gov/ij/). Cell tracking was performed using SIMI-Biocell (SIMI reality motion systems). Origin (OriginLab) and SigmaPlot (Systat Software) were used for statistical analysis, independent *t*-test, and for generating graphs.

Cell-tracking and speed measurements were performed as described previously [77], with minor modifications. SIMI-Biocell (version 4.0 built 155, SIMI reality motion systems) was used for tracking cell movements and for the generation/colouring of 4-D reconstructions. 3-D positions of fluorescently labelled aMpT nuclei were tracked over time manually. 3-D coordinates of the nuclei were saved every 9 minutes (or every 1 minute in Figure 1H and 1H″) during the course of a movie. Cell speeds were measured by calculating the distance moved by aMpT nuclei every 9 minutes (or every 1 minute in Figure 1H and 1H″). Movies and 4-D reconstructions were annotated and represented in their final form using ImageJ (Rasband WS, National Institutes of Health, http://imagej.nih.gov/ij/; 1997–2012).

Cell shape analysis was performed using ImageJ. Single z-slices at 12–18 second intervals were used to manually trace basal cell outlines with the polygon selection tool. Traces were saved using the ROI manager. The centre and total area for each trace was determined with the in-built “centroid” and “area” measurement tools. Cell length was measured by drawing lines through the centroid that connected edges in axes either parallel (distal-proximal) or perpendicular (circumferential) to the distal to proximal tubule length. Area and lengths of a cell were normalised with their average represented as 1.0.

aMpT lengths were calculated by drawing and measuring a segmented line along the distal to proximal tubule length in ImageJ.

### Tip Cell Laser Ablation

Dechorionated *ctB*>*UAS-CD8-GFP* embryos were mounted on double-sided Scotch tape in PBS solution. The TC and surrounding two or three cells were ablated (to ensure removal of both TC and SC) in late 12/early 13 stage embryos. Cell ablation was performed using a 63×/0.9 NA water immersion lens on a Yokogawa spinning disk (CSU-10) confocal microscope fitted with a pulsed nitrogen laser (MicroPoint). Image acquisition and microscope control were by MetaMorph (version 7.0) software (Molecular Devices). Embryos were allowed to develop to stage 16 under humid conditions at 25°C, fixed and processed for immunostaining.

### Antibody Staining and Quantification

Embryos were fixed in 4% paraformaldehyde and devitellinised by vigorous shaking in 1∶1 heptane/methanol. Immunostaining was performed using standard techniques. For pMLC staining (Figure S5), embryos were fixed in 37% formaldehyde for 3–5 minutes and devitellinised using a fine glass needle.

The primary antibodies used were: mouse anti-FasII (1∶10, DSHB); mouse anti-Cut (1∶50, DSHB); rabbit anti ß-gal (1∶10,000, ICN Biomedicals); rabbit anti-Rhomboid (1∶500, gift of E. Bier); rabbit anti-dpERK (1∶50 Cell Signaling technology); rabbit anti-Bazooka (1∶500, gift of A. Wodarz); rabbit anti-Cleaved Caspase3 (1∶20, Cell Signaling technology); goat anti-GFP (1∶500, Abcam); mouse anti-Futsch/22c10 (1∶200, DSHB); rat anti-HA (1∶200, Roche), rabbit anti-phospho-Myosin Light Chain 2 ([Ser19]; 1∶20, Cell Signaling technology).

Secondary antibodies were used at 1∶200. Appropriate biotinylated secondary antibodies were used with the Vector Elite ABC Kit (Vector Laboratories) for DAB staining. FITC- or Cy3-conjugated secondary antibodies were used for fluorescent labelling. When required, streptavidin-conjugated FITC/Cy3 amplification was used. TSA-Biotin amplification system (Perkin-Elmer) was used for dpERK detection. DNA was stained with DAPI (1∶1,000, Molecular Probes). Embryos and tissue were mounted in Vectashield (Vector Laboratories) and viewed on a Leica SP5 confocal microscope.

Image processing was performed using ImageJ and Adobe Photoshop. To measure dpERK staining levels, stage 13 tubules were traced using the segmented line tool in ImageJ with a line width approximately equal to tubule width. The plot profile tool was used to quantify staining intensity along the line. Values were binned into 1 µm bins and averaged for *n* = 7 tubules. Figures were assembled in Adobe Illustrator.

### Semi-thin aMpT Sections

Embryos of the appropriate stage were fixed and stained with anti-FasII, dehydrated, and mounted in Araldite resin. Transverse sections approximately 2.5 µm in thickness were made midway along the distal region of aMpTs using a Reichert microtome.

### Tubule Secretion Assay and Analysis of Adult Fly Osmoregulation

Embryos of the appropriate genotype were collected overnight and aged for a further 6 hours at 29°C. 40 first instar larvae were transferred to a vial of standard food and incubated at 25°C until adults eclosed. Secretory assays were performed as described previously [55] at 23–24°C using 3–5 day old adults. cAMP and LK1 (Sigma) were added to a final concentration of 1 mM and 100 µm at approximately 30 and 60 min, respectively. To measure wet and dry body weights, flies were briefly anaesthetized with CO_2_, transferred to Eppendorf tubes on ice, three flies were pooled and weighed on a Mettler Toledo precision balance (wet weight). The flies were killed by freezing for 20 minutes and transferred to a 50°C oven containing a tray of silica crystals, allowed to desiccate for ∼24 hours, and weighed again (dry weight).

## Supporting Information

Figure S1 **Perturbation of EGF signalling does not affect apicobasal polarity or induce cell death (related to** **Figure 3** **).** (A–C) Stage 15 MpTs stained for Baz (red) and FasII (green) in control (A), *ctB>UAS-EGFR^DN^* (B), and *ctB>UAS-EGFR^act^* (C) embryos. Apical (Baz) and lateral (FasII) markers appear normal under conditions of EGF perturbation. Tubule is outlined in (B, C). (D–F) Stage 15 MpTs stained for cleaved caspase 3 (CC3, red), GFP (green, *ctB>UAS-GAP43-GFP*), and DAPI (blue) in control (D) *ctB>UAS-EGFR^DN^* (E) and *ctB>UAS-EGFR^act^* (F) embryos. Cell death in tubules is not observed in controls or under conditions of EGF perturbation. (G, H) control embryos stained for CC3 (red) and DAPI (blue) showing cell death in the epidermis in a stage 13 embryo (G, higher magnification in G′) and in the central nervous system in a stage 15 embryo (H, higher magnification in H′) demonstrating that CC3 is an effective reporter for cell death.
(TIF)

Figure S2 **Cell shape changes correlated with Myosin II pulses (related to** **Figure 5G** **).** Graphs show normalised basal area (A, B); circumferential (blue) and D-P (black) lengths (A′, B′); and D–P:Circumferential aspect ratio (A″, B″) for two individual representative control cells over time. Periods of Myosin II enrichment are highlighted (green boxes). Observation of individual cells revealed no clear correlation between cell shape and Myosin II pulse versus interpulse periods (*n* = 10 cells).
(TIF)

Figure S3 **Cell shape changes in control and** ***EGFR^DN^*** **and YFP-Zip^DN^ (related to** **Figure 5H and 5I** **).** Normalised basal area, circumferential and D-P lengths in three representative control (A–C), *EGFR^DN^* (A′–C′), and YFP-*Zip^DN^*(A″–C″) cells over time. Shaded areas highlight the extent of fluctuation in measured parameters. Control cells fluctuate more extensively than *EGFR^DN^* and *YFP-Zip^DN^* cells. Polar plot (D) similar to Figure 5I showing centroid displacement in control (green, *n* = 10) and *YFP-Zip^DN^* (yellow, *n* = 4) cells. *YFP-Zip^DN^* cells show reduced speeds of movement compared to controls and remain more closely aligned with the D-P axis of tubules.
(TIF)

Figure S4 **Slam and Myosin-II are not planar polarised in proximal tubule cells (related to** **Figures 4D** **,** **5D, and 5E** **).** (A–A″) Stage 15 MpT stained for Slam-HA (red) and FasII (green). The same MpT as in Figure 4D highlighting the proximal (post-kink) region of the tubule. Slam is not planar polarised as it is in the distal tubule. (B–B″) Basal view of distal (red outline) and proximal (yellow outline) regions of a stage 15 tubule (Movie S14). Arrowheads in (B′) show proximal Myosin II accumulation in a distal cell (B and B″ arrowheads). There is a transient decrease in circumferential cell length during Myosin II accumulation (at times 1∶24 and 1∶48). No Myosin II accumulation is observed in the proximal cells. See also Movie S15.
(TIF)

Figure S5 **Generation of clones of tubule cells expressing EGFR^act^ (related to** **Figures 2F** **and** **4A–4D** **).** One cell of a two-cell clone (expressing the constitutively active EGFR^act^; GFP in green) is visible in a tubule that has been stained with FasII to highlight cell boundaries and phospho-Myosin Light Chain (pMLC) to analyse cortical distribution of phosphorylated Myosin II. At this particular z-plane there are no Myosin II crescents in mutant or wild type cells but we found several proximal crescents in wild type cells in different z-planes (in which the clone was not visible). Asterisk, TC.
(DOCX)

Table S1 **The table lists the PCP alleles analysed, whether maternal (M), zygotic (Z), or both (M/Z) contributions were removed and their effects on MpT C–E and Slam-HA localisation.** Images of representative embryos are shown below the table.
(DOC)

Data S1 **Raw data supporting graphical figures and charts.**
(XLSX)

Movie S1 **z-projection showing aMpT elongation over 6 hours (related to** **Figure 1C** **).** *ctB*>*StingerRed* embryo (white) labels aMpT nuclei. Part of the posterior MpT (pMpT) can be seen to the right from 60 min onwards. Embryonic aMpTs with anterior to the left and dorsal at the top.
(MOV)

Movie S2 **SIMI-Biocell assisted 4-D reconstruction of aMpT distal region (right panel) from aMpT shown on the left (related to** **Figure 1F** **).** Spheres mark position of nuclei; TC is shown by a star. Spheres were coloured arbitrarily at 197∶15 min to discern pattern of cell rearrangements. Embryonic aMpTs with anterior to the left and dorsal at the top.
(MOV)

Movie S3 **Reconstructed tubule shown in movie 2 at 0∶00 min to show arrangement of cells around the tubule lumen at the beginning of elongation process (related to** **Figure 1G** **).** Two adjacent rings of cells are marked in white and black; star indicates the TC at the distal end. Embryonic aMpTs with anterior to the left and dorsal at the top.
(MOV)

Movie S4 **Cells in the top plane of an aMpT are shown in different colours. Intercalation of cells between their neighbours can be followed (related to Figure IH).** Arrows in Fig. 1H-H′′ indicate one such intercalating cell (green). Embryonic aMpTs with anterior to the left and dorsal at the top.
(MOV)

Movie S5 **z-projection showing an aMpT in a** ***ctB>StingerRed*** **,** ***EGFR^DN^*** **embryo over 6 hours (related to** **Figure 3D** **).** Embryonic aMpTs with anterior to the left and dorsal at the top.
(MOV)

Movie S6 **4-D reconstruction (right) of 42 distal cells in a** ***ctB>StingerRed*** **,** ***EGFR^DN^*** **aMpT (left) (related to** **Figure 3D** **).** Cells were coloured in circumferential rows; TC is indicated by a star. Embryonic aMpTs with anterior to the left and dorsal at the top.
(MOV)

Movie S7 **A 6 hour movie showing z-projection of aMpT elongation in a** ***ctB>StingerRed*** **,** ***EGFR^act^*** **embryo (related to** **Figure 3F** **).** Embryonic aMpTs with anterior to the left and dorsal at the top.
(MOV)

Movie S8 **Distal region 4-D reconstuction (right) of** ***ctB>StingerRed*** **,** ***EGFR^act^*** **aMpT (left) (related to Figure 3F).** 38 cells coloured circumferentally are shown. Embryonic aMpTs with anterior to the left and dorsal at the top.
(MOV)

Movie S9 **Stage 14** ***sqh^AX3^*** **;** ***sqh-Sqh::GFP*** **;** ***sqh-Sqh::GFP*** **aMpT showing Myosin II/Sqh::GFP (white) dynamics in the basal cortex of ∼10 distal aMpT cells (related to** **Figure 5D** **).** Arrow at 1∶28 min indicates cell shown in Movie 10. Embryonic aMpTs with anterior to the left and dorsal at the top.
(MOV)

Movie S10 **Enlarged view of a single cell from Movie S9 showing Myosin II/Sqh::GFP (white) pulses in the proximal cortex (related to** **Figure 5D** **).** Time (min) is shown. Embryonic aMpTs with anterior to the left and dorsal at the top.
(MOV)

Movie S11 ***ctB>GAP43::GFP*** **labelled membrane (left, magenta in merge) and Myosin II/** ***sqh-Sqh::mCherry*** **(middle, green in merge) dynamics in a stage 14 control aMpT over 10 min (related to** **Figure 5E** **).** Embryonic aMpTs with anterior to the left and dorsal at the top.
(MOV)

Movie S12 **Membrane (left, magenta in merge) and Myosin II (middle, green in merge) in a stage 14** ***ctB>EGFR^DN^*** **,** ***GAP43::GFP*** **,** ***sqh-Sqh::mCherry*** **embryo (related to** **Figure 5F** **).** Embryonic aMpTs with anterior to the left and dorsal at the top.
(MOV)

Movie S13 **Basal membrane dynamics in a stage 14** ***ctB>YFP-Zip^DN^*** **,** ***GAP43::GFP*** **embryo over 12 minutes (related to** **Figure 5G** **and Figure S3A″–C″).** Arrow indicates a cell with basal, proximal YFP-*Zip^DN^* accumulation that persists throughout the 12 minute period. Cell outlines are marked with GAP43::GFP. Embryonic aMpTs with anterior to the left and dorsal at the top.
(MOV)

Movie S14 **A live stage 15 tubule with membrane (left panels,** ***ctB>GAP43::GFP*** **) and Myosin II (middle panels,** ***sqh-Sqh::mCherry*** **) shows absence of polarised Myosin II pulses in proximal cells (related to Figure S4B).** See Figure S4B for details. Embryonic aMpTs with anterior to the left and dorsal at the top.
(MOV)

Movie S15 **Lateral view of an aMpT in a stage 15** ***sqhAX3; sqh-Sqh::GFP; sqh-Sqh::GFP*** **embryo (related to** **Figures 5D, 5E** **, and S4B).** Distal (red) and proximal (yellow) tubule regions outlined. Arrowhead indicates a TC. A single z-plane imaged for 5.7 minutes shows highly dynamic Myosin II (white) pulses in the distal regions but no Myosin II activity proximally. Embryonic aMpTs with anterior to the left and dorsal at the top.
(MOV)

## Abbreviations

aMpT: Anterior Malpighian tubule
CE: convergent extension
dpERK: diphosphosphorylated extracellular signal-regulated kinase
D-P: distal-proximal
EGF: epidermal growth factor
EGFR: epidermal growth factor receptor
Fas II: fasciclin II
MpT: Malpighian tubule
PCP: planar cell polarity
PC: principal cell
RTK: receptor tyrosine kinase
SC: sibling cell
Spi: Spitz
sSpi: secreted Spitz
Sqh: spaghetti squash
TC: tip cell
