# Supplementary figures and images for: Epidermal Growth Factor Signalling Controls Myosin II Planar Polarity to Orchestrate Convergent Extension Movements during Drosophila Tubulogenesis
Source: PLoS Biol. 2014 Dec 2;12(12):e1002013. doi: 10.1371/journal.pbio.1002013 (PMC4251826; doi:10.1371/journal.pbio.1002013)

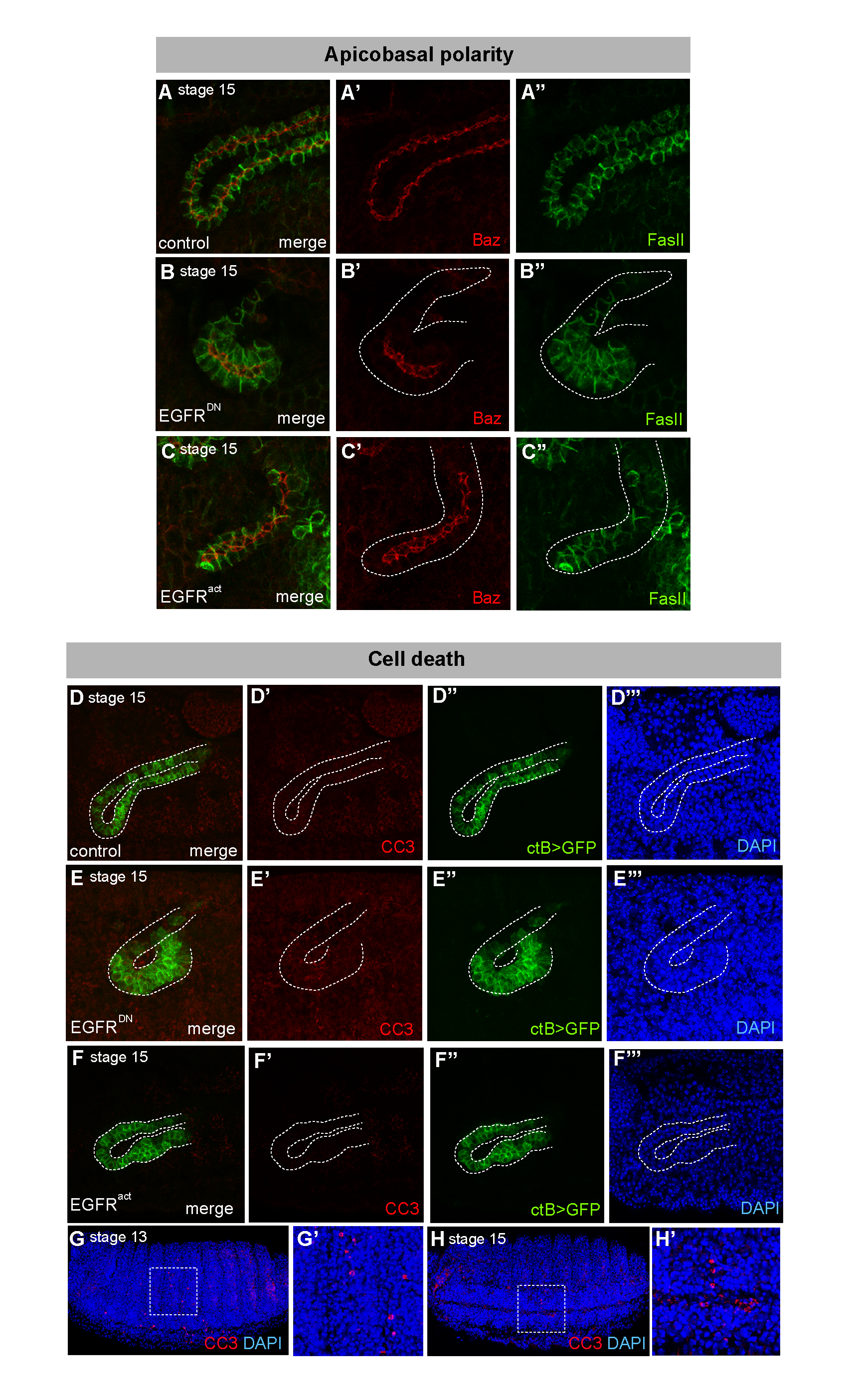

Supplement: Figure S1 — Perturbation of EGF signalling does not affect apicobasal polarity or induce cell death (related to Figure 3 ). (A–C) Stage 15 MpTs stained for Baz (red) and FasII (green) in control (A), ctB>UAS-EGFRDN (B), and ctB>UAS-EGFRact (C) embryos. Apical (Baz) and lateral (FasII) markers appear normal under conditions of EGF perturbation. Tubule is outlined in (B, C). (D–F) Stage 15 MpTs stained for cleaved caspase 3 (CC3, red), GFP (green, ctB>UAS-GAP43-GFP), and DAPI (blue) in control (D) ctB>UAS-EGFRDN (E) and ctB>UAS-EGFRact (F) embryos. Cell death in tubules is not observed in controls or under conditions of EGF perturbation. (G, H) control embryos stained for CC3 (red) and DAPI (blue) showing cell death in the epidermis in a stage 13 embryo (G, higher magnification in G′) and in the central nervous system in a stage 15 embryo (H, higher magnification in H′) demonstrating that CC3 is an effective reporter for cell death. (TIF) [file pbio.1002013.s001.tif]

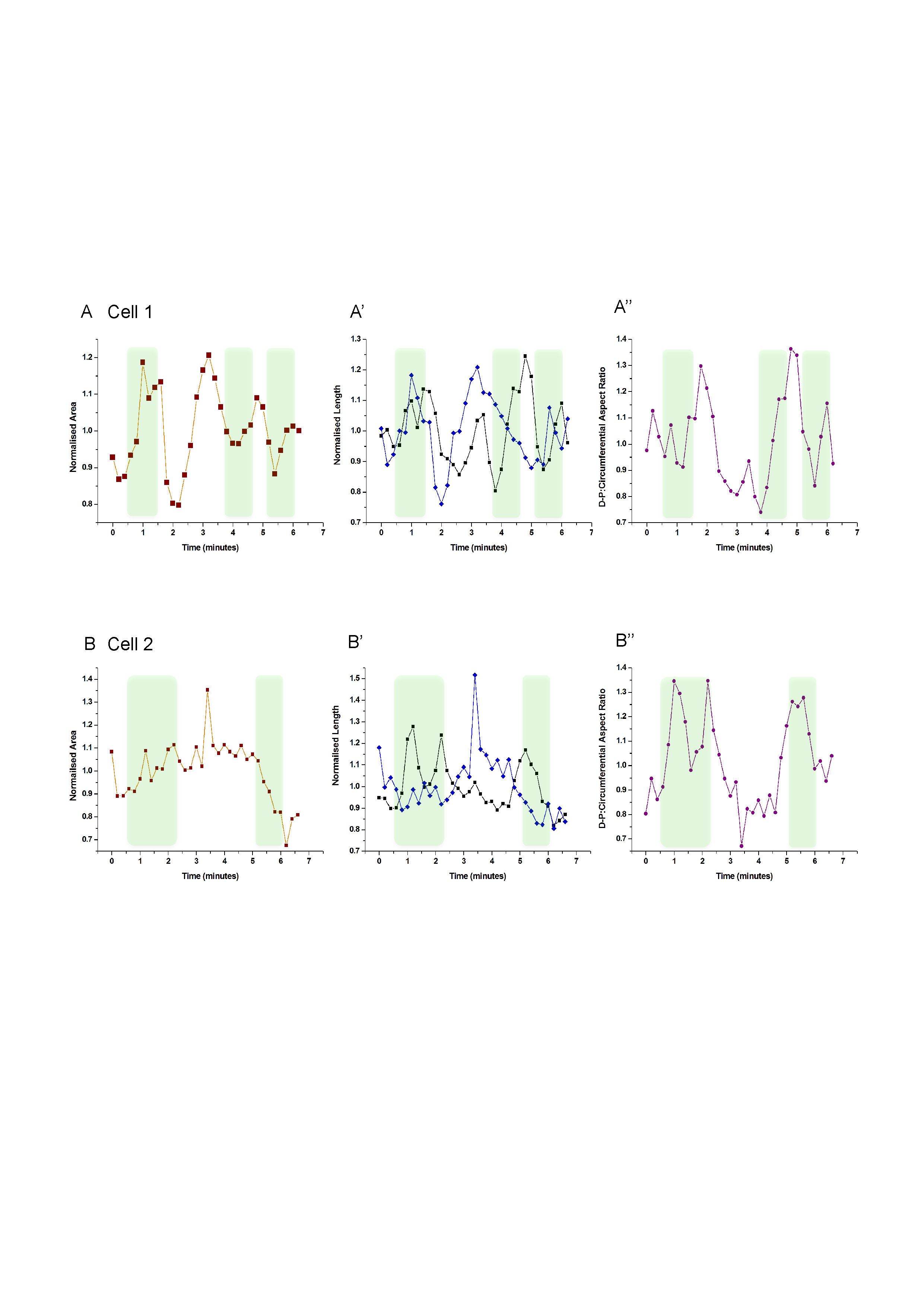

Supplement: Figure S2 — Cell shape changes correlated with Myosin II pulses (related to Figure 5G ). Graphs show normalised basal area (A, B); circumferential (blue) and D-P (black) lengths (A′, B′); and D–P:Circumferential aspect ratio (A″, B″) for two individual representative control cells over time. Periods of Myosin II enrichment are highlighted (green boxes). Observation of individual cells revealed no clear correlation between cell shape and Myosin II pulse versus interpulse periods (n = 10 cells). (TIF) [file pbio.1002013.s002.tif]

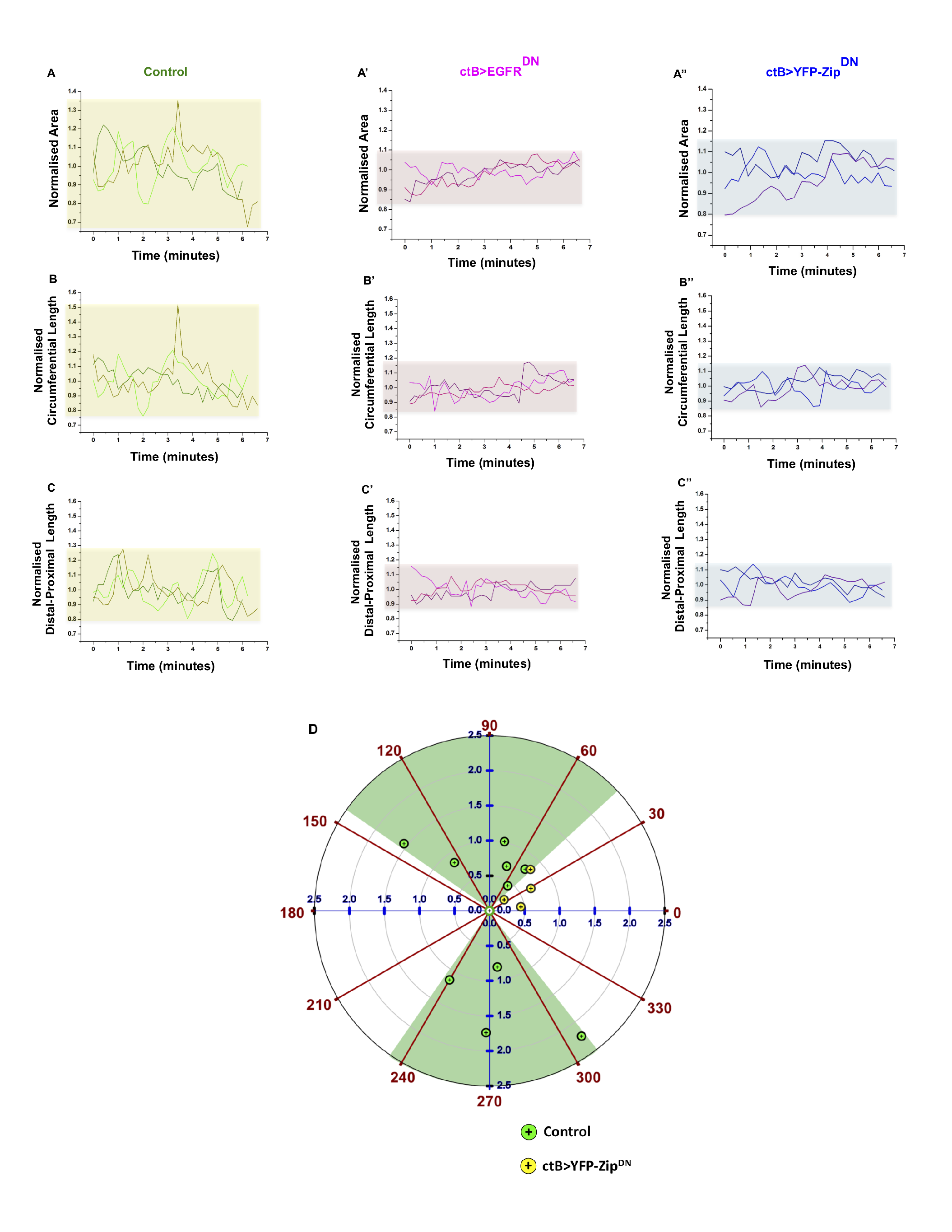

Supplement: Figure S3 — Cell shape changes in control and EGFRDN and YFP-ZipDN (related to Figure 5H and 5I ). Normalised basal area, circumferential and D-P lengths in three representative control (A–C), EGFRDN (A′–C′), and YFP-ZipDN(A″–C″) cells over time. Shaded areas highlight the extent of fluctuation in measured parameters. Control cells fluctuate more extensively than EGFRDN and YFP-ZipDN cells. Polar plot (D) similar to Figure 5I showing centroid displacement in control (green, n = 10) and YFP-ZipDN (yellow, n = 4) cells. YFP-ZipDN cells show reduced speeds of movement compared to controls and remain more closely aligned with the D-P axis of tubules. (TIF) [file pbio.1002013.s003.tif]

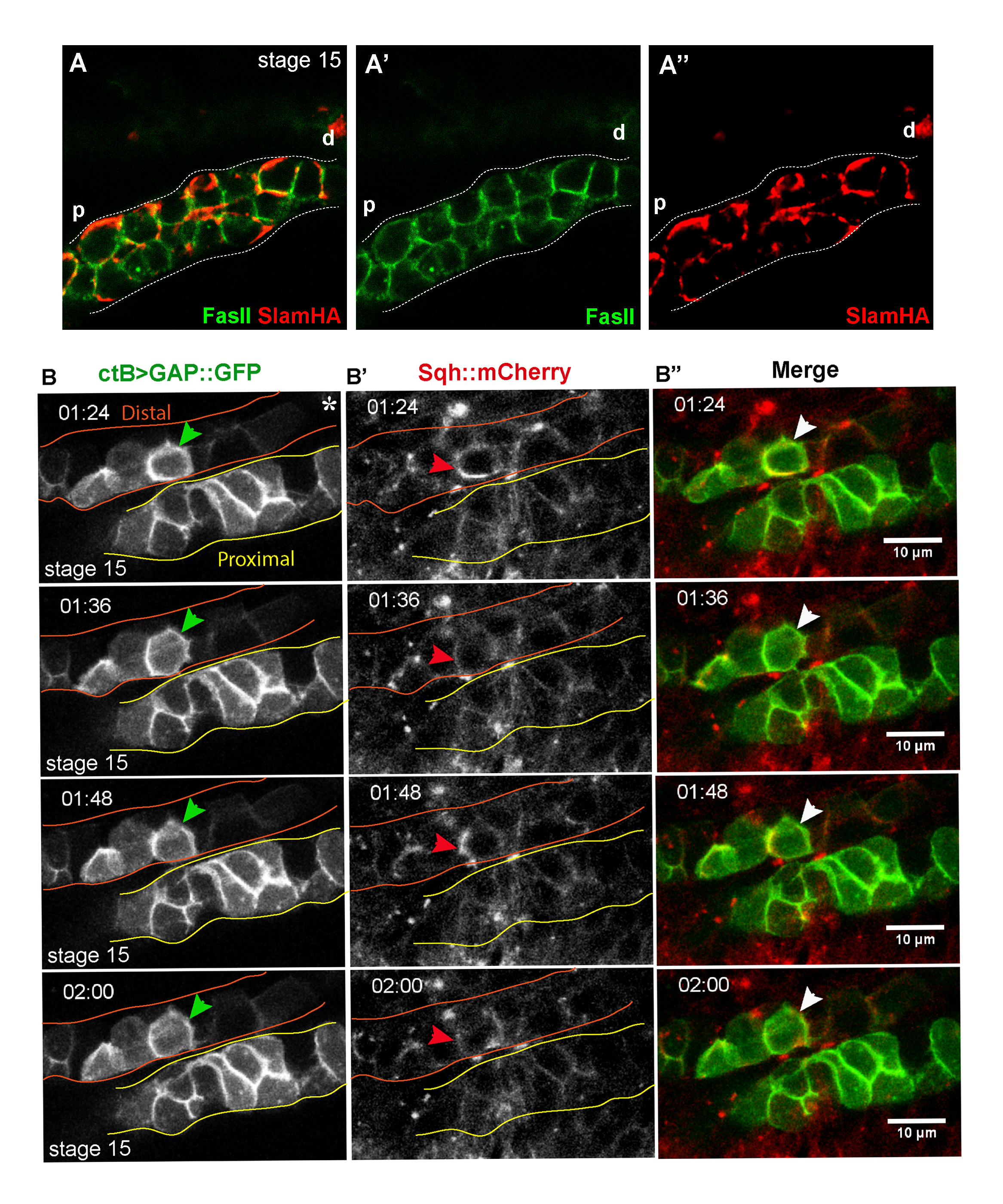

Supplement: Figure S4 — Slam and Myosin-II are not planar polarised in proximal tubule cells (related to Figures 4D , 5D, and 5E ). (A–A″) Stage 15 MpT stained for Slam-HA (red) and FasII (green). The same MpT as in Figure 4D highlighting the proximal (post-kink) region of the tubule. Slam is not planar polarised as it is in the distal tubule. (B–B″) Basal view of distal (red outline) and proximal (yellow outline) regions of a stage 15 tubule (Movie S14). Arrowheads in (B′) show proximal Myosin II accumulation in a distal cell (B and B″ arrowheads). There is a transient decrease in circumferential cell length during Myosin II accumulation (at times 1∶24 and 1∶48). No Myosin II accumulation is observed in the proximal cells. See also Movie S15. (TIF) [file pbio.1002013.s004.tif]
