# Supplementary material for: Epidermal Growth Factor Signalling Controls Myosin II Planar Polarity to Orchestrate Convergent Extension Movements during Drosophila Tubulogenesis
Source: PLoS Biol. 2014 Dec 2;12(12):e1002013. doi: 10.1371/journal.pbio.1002013 (PMC4251826; doi:10.1371/journal.pbio.1002013)
Supplement: Figure S5 — Generation of clones of tubule cells expressing EGFRact (related to Figures 2F and 4A–4D ). One cell of a two-cell clone (expressing the constitutively active EGFRact; GFP in green) is visible in a tubule that has been stained with FasII to highlight cell boundaries and phospho-Myosin Light Chain (pMLC) to analyse cortical distribution of phosphorylated Myosin II. At this particular z-plane there are no Myosin II crescents in mutant or wild type cells but we found several proximal crescents in wild type cells in different z-planes (in which the clone was not visible). Asterisk, TC. (DOCX) [file pbio.1002013.s005.docx]

**Supplementary Figure 5**

*
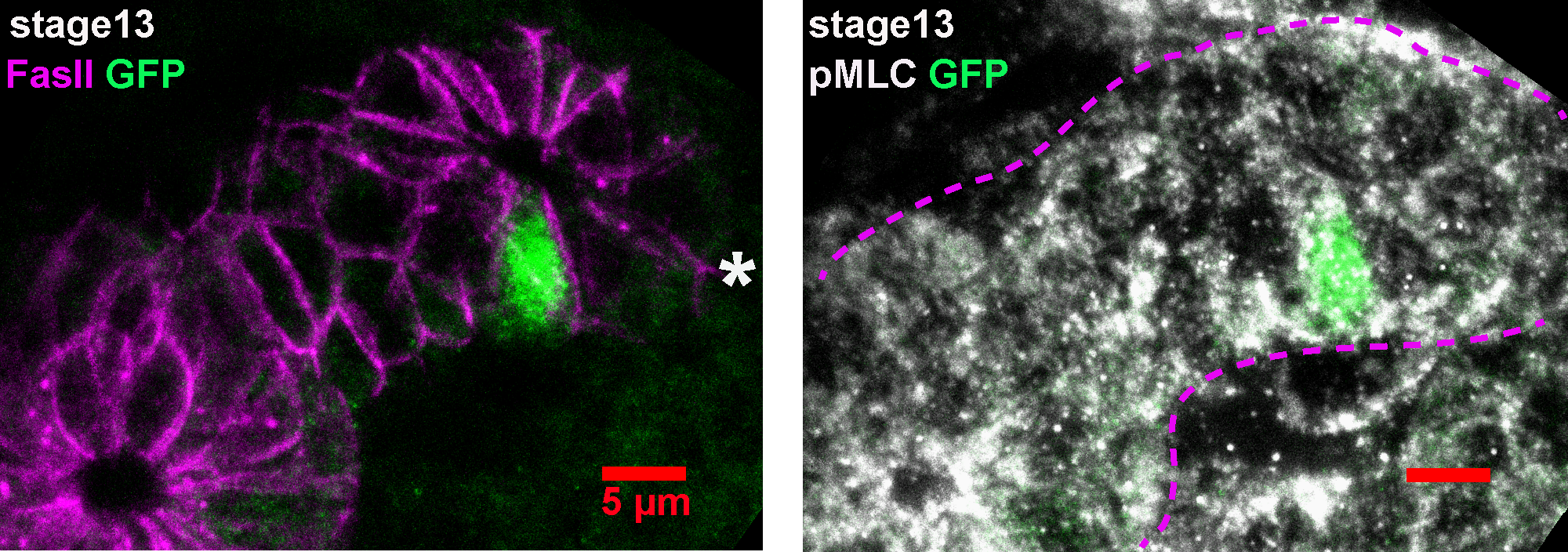
*

**Generation of clones of tubule cells lacking EGF signalling.**

We have made clones of marked tubule cells using cis-FLP-out recombination and tested multiple protocols to identify the most favourable times and conditions for clone induction. Even if we induce recombination early in embryogenesis (during syncytial stages) we find clones only rarely in the tubules and they are never larger than 2 or 3 cells. We successfully generated clones with the following genotypes: EGFR^DN^, activated EGF receptor (EGFR^act^, above) and expressing sSpi. We have examined more than 150 embryos and analysed all the resulting clones - 4 in all, 2 in each of EGFR^DN^ & EGFR^act^  (sSpi clones proved uninterpretable due to a leaky GFP artefact).

We stained fixed preparations for phosphoMyosin to analyse its cortical distribution as a functional readout of polarity. As expected, we were unable to detect Myosin crescents within clones of either mutant genotype, while we saw the normal proximal crescents in wild type cells outside the clones. However the clones themselves are so small that it is not feasible to draw any conclusions about non-cell autonomous effects on cells neighbouring clones, as such small clones never create a break in the continuity of wild type cells along the tubule D-P axis.
