# Supplementary material for: Epidermal Growth Factor Signalling Controls Myosin II Planar Polarity to Orchestrate Convergent Extension Movements during Drosophila Tubulogenesis
Source: PLoS Biol. 2014 Dec 2;12(12):e1002013. doi: 10.1371/journal.pbio.1002013 (PMC4251826; doi:10.1371/journal.pbio.1002013)
Supplement: Table S1 — The table lists the PCP alleles analysed, whether maternal (M), zygotic (Z), or both (M/Z) contributions were removed and their effects on MpT C–E and Slam-HA localisation. Images of representative embryos are shown below the table. (DOC) [file pbio.1002013.s006.doc]

­­­

| Genotype | Maternal (M)/ zygotic (Z) | Convergent extension defects | Slam localisation |
| --- | --- | --- | --- |
| *dsh1* | M/Z | none | - |
| *fz1* | M/Z | none | - |
| *stbm6* | M/Z | none | - |
| *dsUAO71* | M/Z | none | - |
| *dsUAO71, stanE59* | Z | none | normal |
| *dsUAO71, stan3* | Z | none | - |
| *ftG-rv* | Z | none | - |


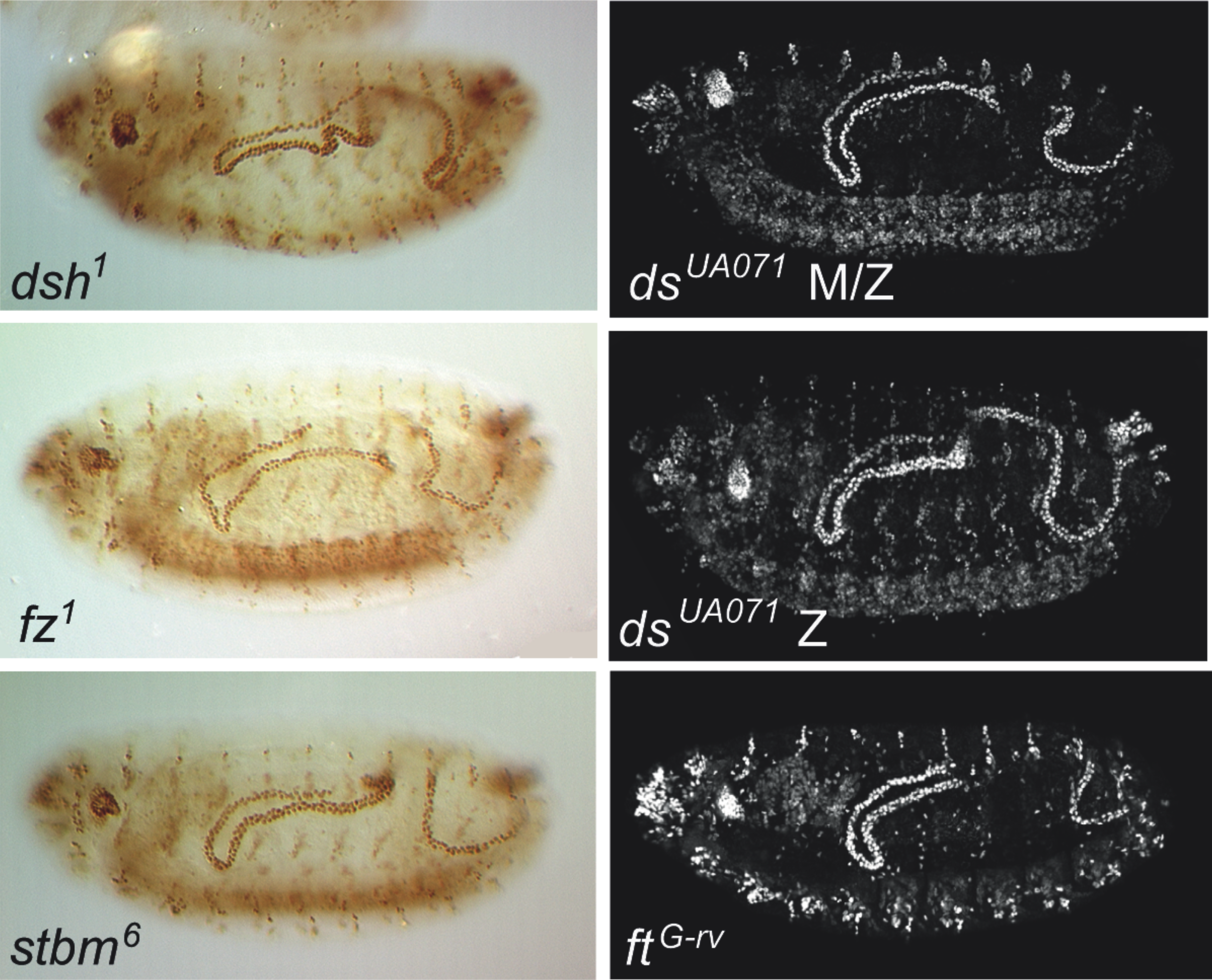
­
